# Supplementary figures and images for: Microbial colonization induces histone acetylation critical for inherited gut-germline-neural signaling
Source: PLoS Biol. 2021 Mar 31;19(3):e3001169. doi: 10.1371/journal.pbio.3001169 (PMC8041202; doi:10.1371/journal.pbio.3001169)

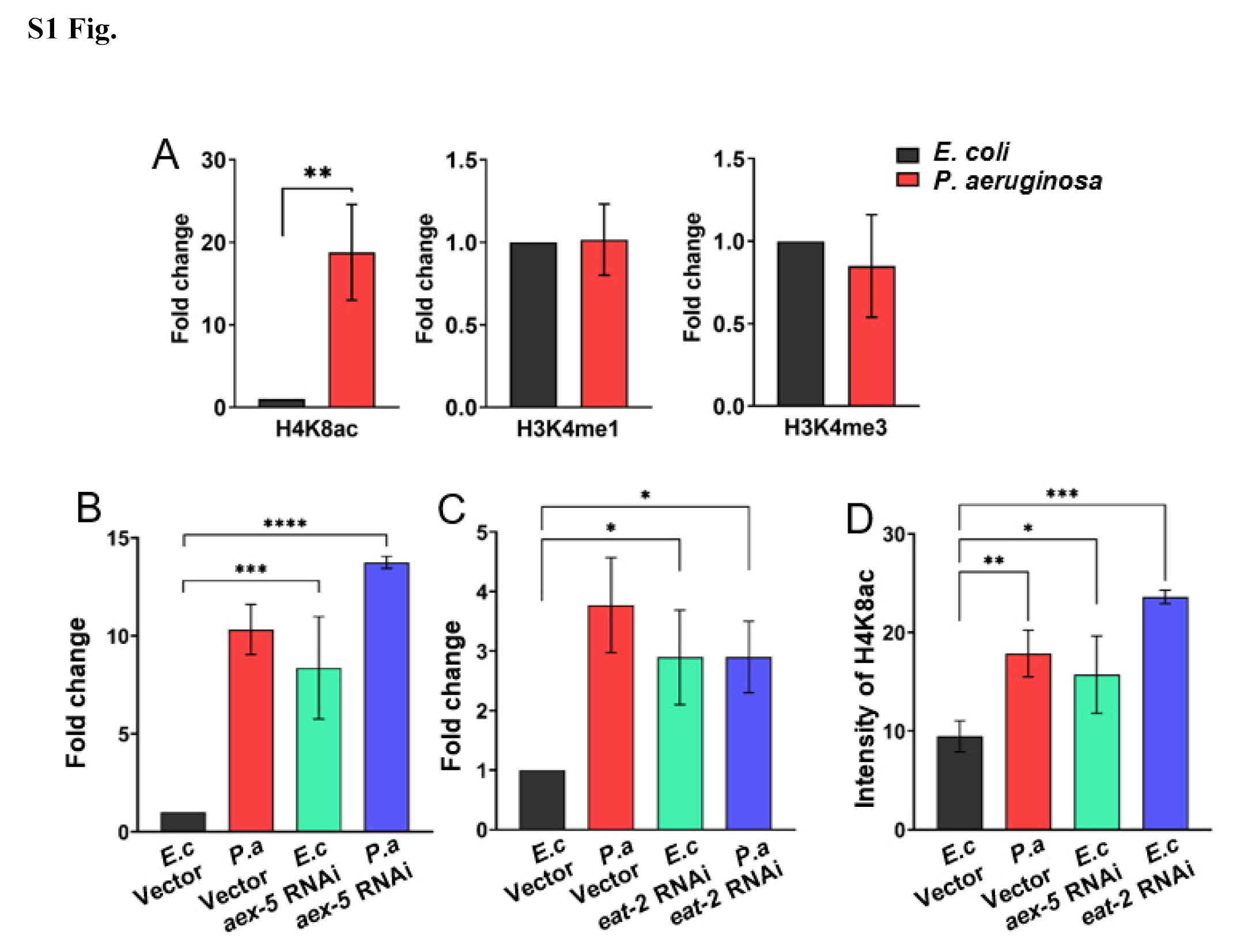

Supplement: S1 Fig — (A) Quantification of band density of western blots assay performed on fer-1(b232) animals exposed to E. coli (E. c) or P. aeruginosa (P. a) for 24 hours at 25°C (n ≈ 1,000). (B) Quantification of band density of western blots assay performed on fer-1(b232) animals exposed to E. coli (E. c) or P. aeruginosa (P. a) for 24 hours at 25°C following aex-5 (n ≈ 1,000) and (C) eat-2 RNAi (n ≈ 1,000). Chemiluminescence signals from samples were detected, and the densities of the protein bands were quantified and represented as fold change. Fold change is the ratio of mean density of a given sample over the control E. coli sample or the control E. coli vector sample for the RNAi assays. The fer-1(b232) animals were maintained at 15°C. To induce sterility, L1-stage animals were transferred to 25°C and allowed to develop. L4-stage animals were then transferred to RNAi plates and allowed to grow for 24 hours at 25°C. Pathogen exposure was performed at 25°C for 24 hours. (D) Quantification of immunofluorescence of wild-type N2 animals stained with anti-H4K8ac antibody post exposure to E. coli (E. c) or P. aeruginosa (P. a) for 24 hours at 25°C following aex-5 and eat-2 RNAi (n = 5). Three independent experiments were performed for the above experiments (A–D, except for H3K4me3 immunoblot assay). “n” represents the number of animals for each experiment. “*” asterisk indicates significant difference; *P ≤ 0.05, **P ≤ 0.005, ***P ≤ 0.0005, ****P ≤ 0.0001. See S1 Data for the corresponding data. H4K8ac, histone H4 Lys8 acetylation; H3K4me1, monomethylation of histone H3 Lys4; H3K4me3, trimethylation of histone H3 Lys4; RNAi, RNA interference. (TIF) [file pbio.3001169.s001.tif]

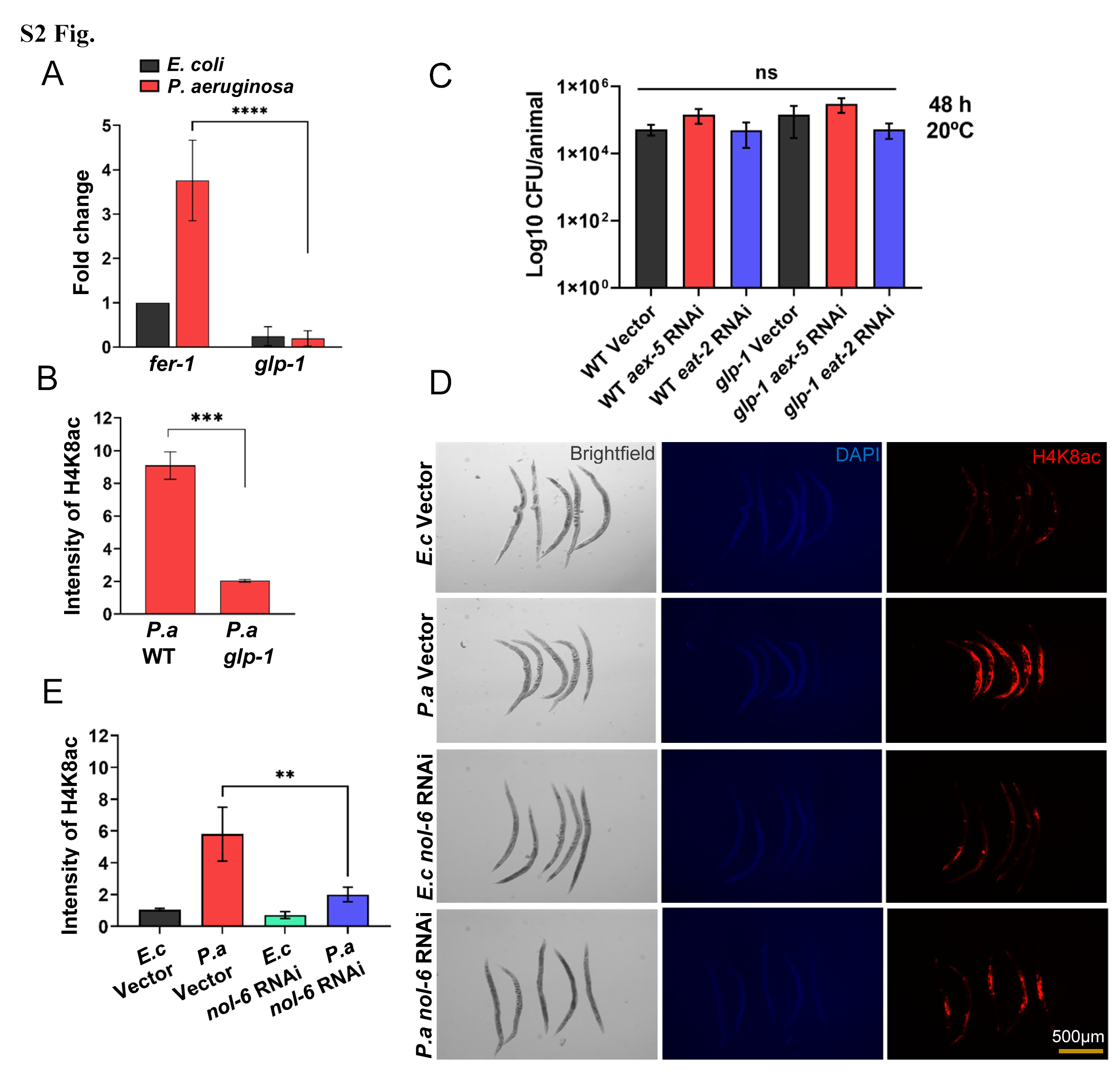

Supplement: S2 Fig — (A) Quantification of band density of western blots assay from fer-1(b232) and glp-1(e2141) animals exposed to E. coli or P. aeruginosa for 24 hours at 25°C (n ≈ 1,000). Four independent experiments were performed. The densities of the protein bands were quantified and represented as fold change. Fold change is the ratio of mean density of a given sample over the control fer-1 E. coli sample. (B) Quantification of immunofluorescence of wild-type N2 and glp-1(e2141) animals stained with anti-H4K8ac antibody after exposure to P. aeruginosa (P. a) for 24 hours at 25°C (n = 5). (C) CFU of wild-type N2 or glp-1(e2141) animals grown on vector control, aex-5 RNAi, or eat-2 RNAi were exposed to P. aeruginosa-GFP for 48 hours at 20°C. Bars represent mean log10 CFU ± SEM. The fer-1(b232) and glp-1(e2141) animals were maintained at 15°C. To induce sterility, L1 animals were transferred to 25°C and allowed to develop. L4 animals were then transferred to RNAi plates and allowed to grow for 24 hours at 25°C. Pathogen exposure was performed at 25°C for 24 hours, unless otherwise indicated. (D) Representative microscopic images of wild-type N2 animals treated with vector control or nol-6 RNAi and stained with anti-H4K8ac antibody following exposure to E. coli (E. c) or P. aeruginosa (P. a) for 24 hours at 25°C. (E) Quantification of immunofluorescence of wild-type N2 animals stained with anti-H4K8ac antibody post exposure to E. coli (E. c) or P. aeruginosa (P. a) for 24 hours at 25°C following nol-6 RNAi (n = 5). Three independent experiments were performed for the above experiments (B–E). “n” represents the number of animals for each experiment. “ns” indicates nonsignificant; “*” asterisk indicates significant difference; *P ≤ 0.05, **P ≤ 0.005, ***P ≤ 0.0005, ****P ≤ 0.0001. See S1 Data for the corresponding data. CFU, colony-forming unit; GFP, green fluorescent protein; H4K8ac, histone H4 Lys8 acetylation; RNAi, RNA interference; WT, wild-type. (TIF) [file pbio.3001169.s002.tif]

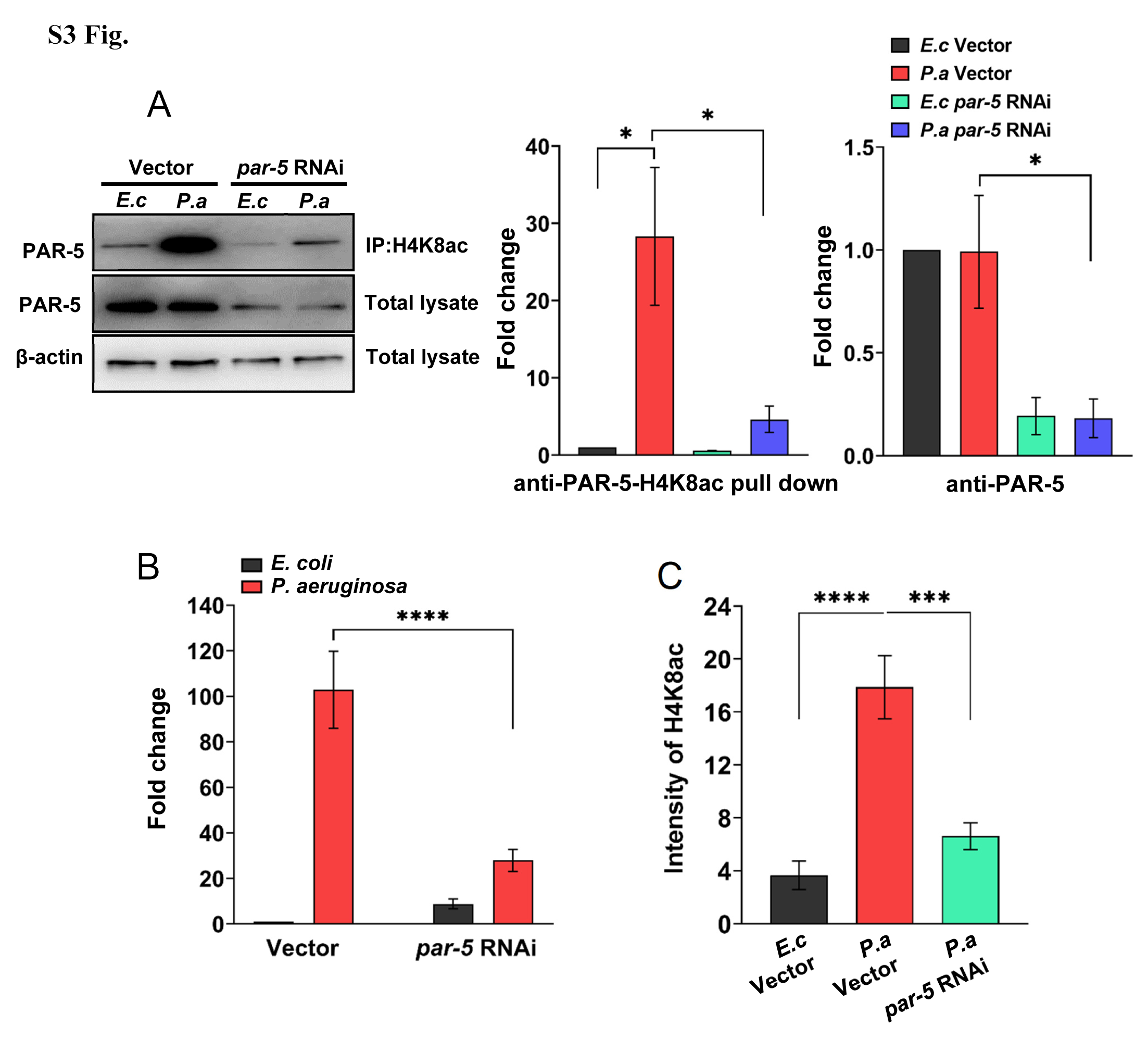

Supplement: S3 Fig — (A) Coimmunoprecipitation of PAR-5 using anti-H4K8ac antibody followed by western blot detection of PAR-5 on fer-1(b232) animals exposed to E. coli (E. c) or P. aeruginosa (P. a) for 24 hours at 25°C following par-5 RNAi (n ≈ 2,000). Error bar represents ±SEM. Three independent experiments were performed. (B) Quantification of band density of western blots assay performed on fer-1(b232) animals exposed to E. coli or P. aeruginosa for 24 hours at 25°C following par-5 RNAi (n ≈ 1,000). Three independent experiments were performed. Chemiluminescence signals from samples were detected; the densities of the protein bands were quantified and represented as fold change. Fold change is the ratio of mean density of a given sample over the control E. coli vector sample. (C) Quantification of the immunofluorescence of wild-type N2 animals stained with anti-H4K8ac antibody after exposure to E. coli (E. c) or P. aeruginosa (P. a) following par-5 RNAi (n = 5). Three independent experiments were performed. “n” represents the number of animals for each experiment. “*” asterisk indicates significant difference; *P ≤ 0.05, ***P ≤ 0.0005, ****P ≤ 0.0001. See S1 Raw Images for uncropped immunoblot images and S1 Data for the corresponding data. H4K8ac, histone H4 Lys8 acetylation; RNAi, RNA interference. (TIF) [file pbio.3001169.s003.tif]

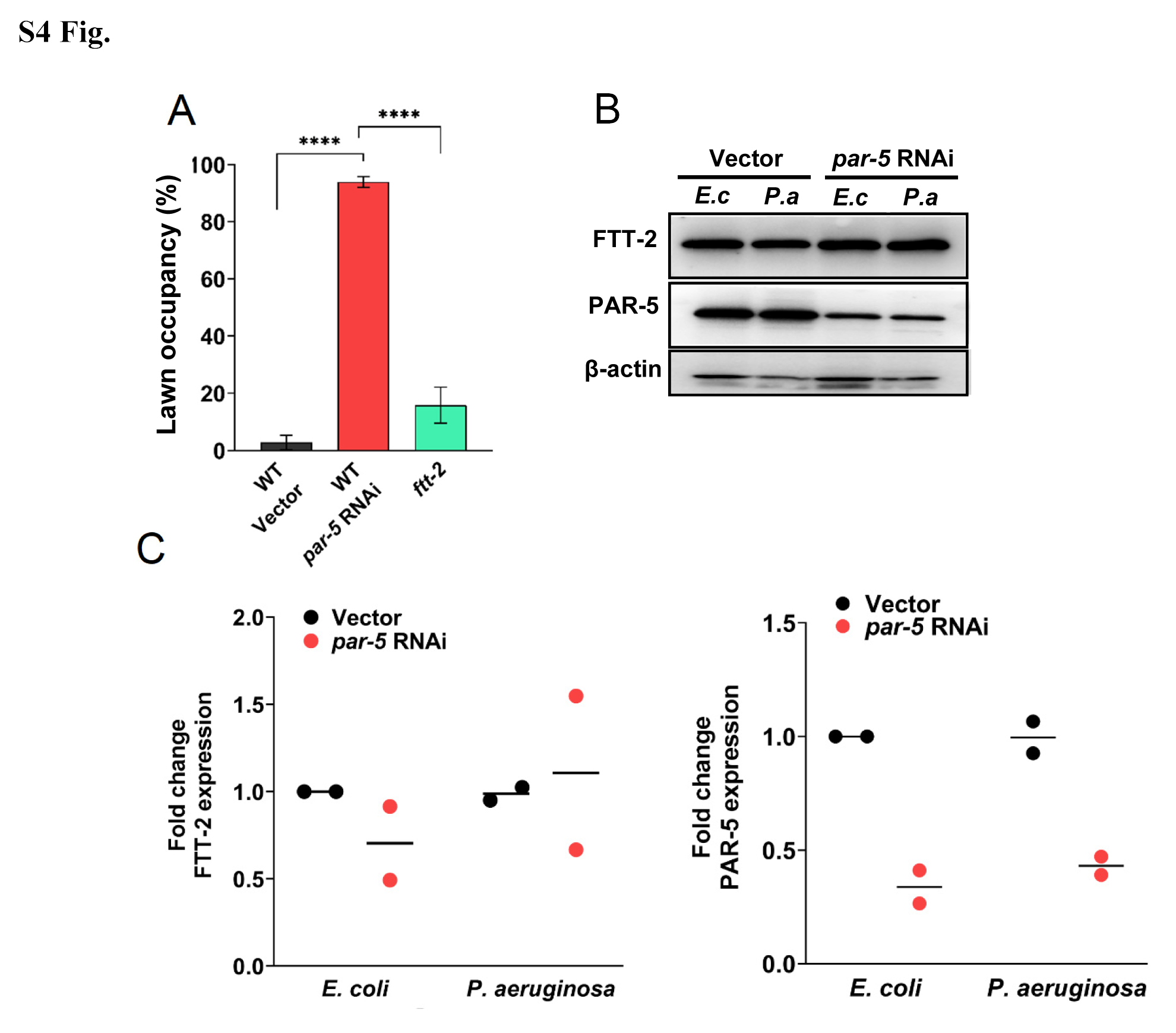

Supplement: S4 Fig — (A) Lawn occupancy of wild-type N2 or ftt-2(n4426) animals at 24 hours following par-5 RNAi at 25°C (n = 20). Three independent experiments were performed. (B) Western blot detection of FTT-2 or PAR-5 and (C) its quantification on extracts of fer-1(b232) animals, exposed to E. coli (E.c) or P. aeruginosa (P.a) for 24 hours at 25°C following par-5 RNAi (n ≈ 1,000). Two independent experiments were performed. Chemiluminescence signals from samples were detected, and the densities of the protein bands were quantified and represented as fold change. Fold change is the ratio of mean density of a given sample over the control E. coli vector sample. The fer-1(b232) animals were maintained at 15°C. To induce sterility, L1-stage animals were transferred to 25°C and allowed to develop. L4-stage animals were then transferred to RNAi plates and allowed to grow for 24 hours at 25°C. “n” represents the number of animals for each experiment. “*” asterisk indicates significant difference; ****P ≤ 0.0001. See S1 Raw Images for uncropped immunoblot images and S1 Data for the corresponding data. RNAi, RNA interference; WT, wild-type. (TIF) [file pbio.3001169.s004.tif]

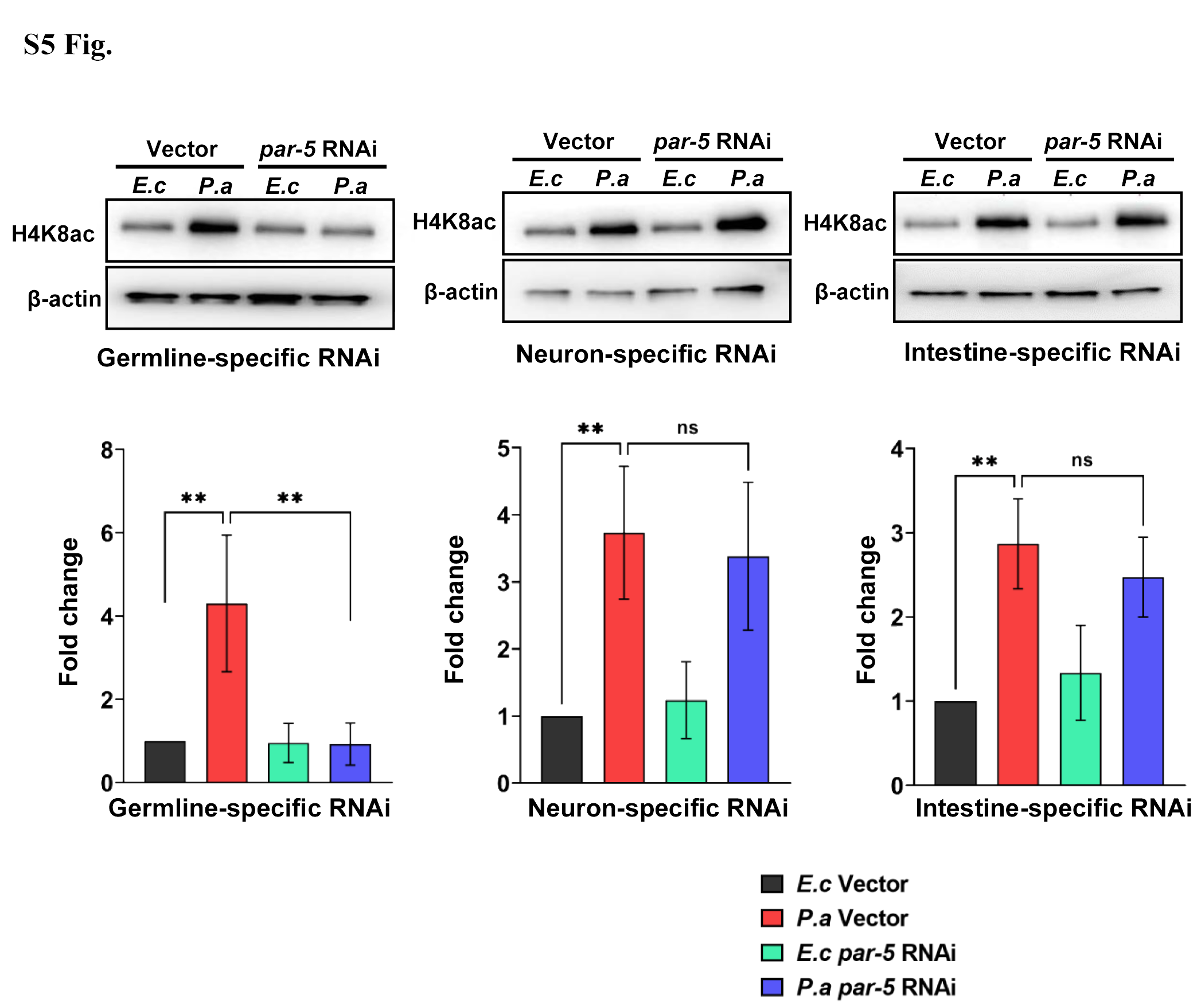

Supplement: S5 Fig — Western blot detection and quantification of H4K8ac on different tissue-specific RNAi animals upon par-5 RNAi and subsequent exposure to E. coli (E. c) or P. aeruginosa (P. a) for 24 hours at 25°C (n ≈ 1,000). Three independent experiments were performed. Chemiluminescence signals from samples were detected, and the densities of the protein bands were quantified and represented as fold change. Fold change is the ratio of mean density of a given sample over the control E. coli vector sample. “n” represents the number of animals for each experiment. “ns” indicates nonsignificant; “*” asterisk indicates significant difference; **P ≤ 0.005. See S1 Raw Images for uncropped immunoblot images and S1 Data for the corresponding data. H4K8ac, histone H4 Lys8 acetylation; RNAi, RNA interference. (TIF) [file pbio.3001169.s005.tif]

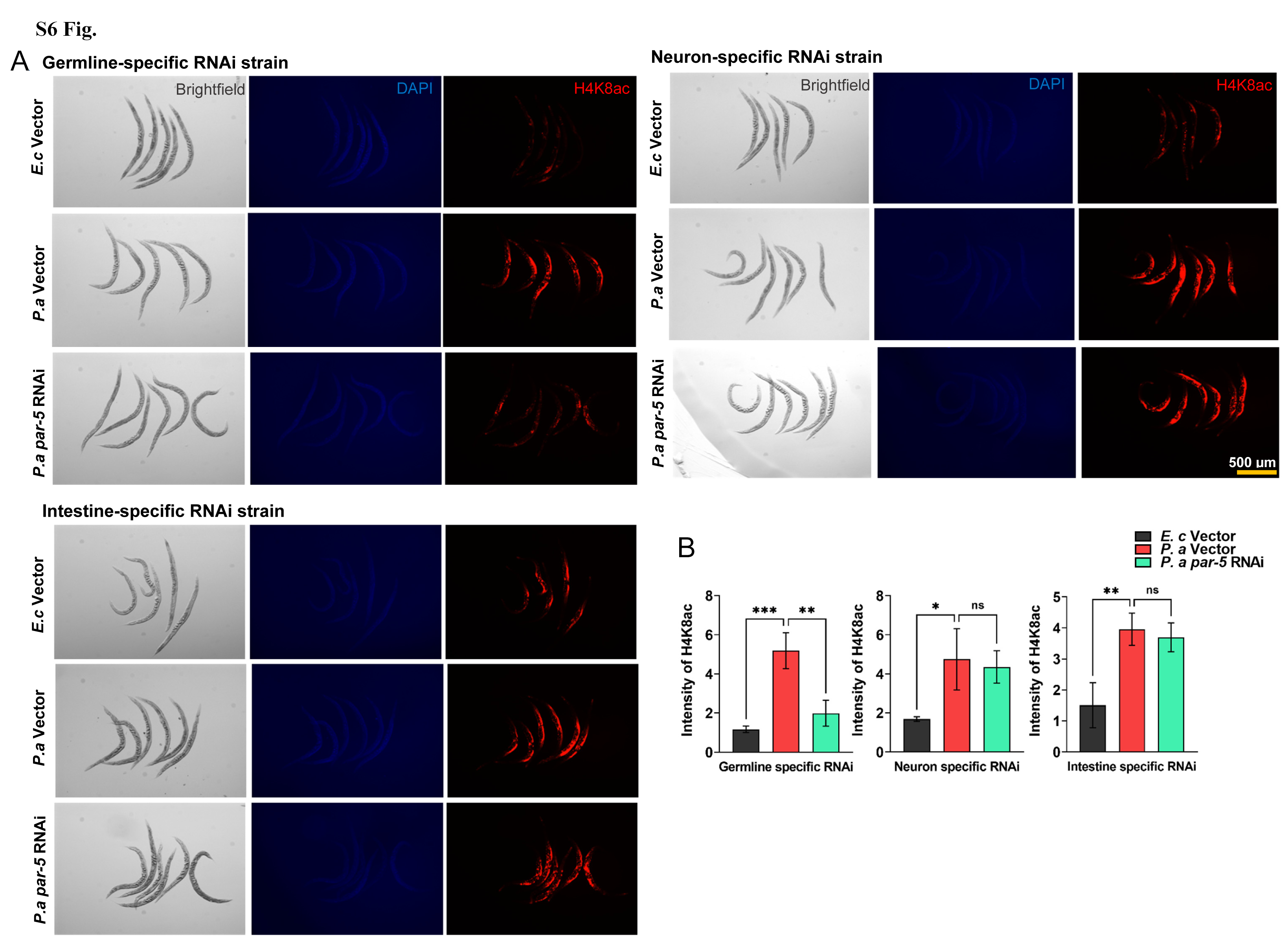

Supplement: S6 Fig — (A) Whole-mount immunofluorescence profiles of tissue-specific RNAi animals stained with anti-H4K8ac antibody. (B) Quantification of immunofluorescence of tissue-specific RNAi animals exposed to E. coli (E. c) or P. aeruginosa (P. a) for 24 hours at 25°C, following par-5 RNAi induction for 24 hours (n = 5). Three independent experiments were performed. “n” represents the number of animals for each experiment. “ns” indicates nonsignificant; “*” asterisk indicates significant difference; *P ≤ 0.05, **P ≤ 0.005, ***P ≤ 0.0005. See S1 Data for the corresponding data. H4K8ac, histone H4 Lys8 acetylation; RNAi, RNA interference. (TIF) [file pbio.3001169.s006.tif]

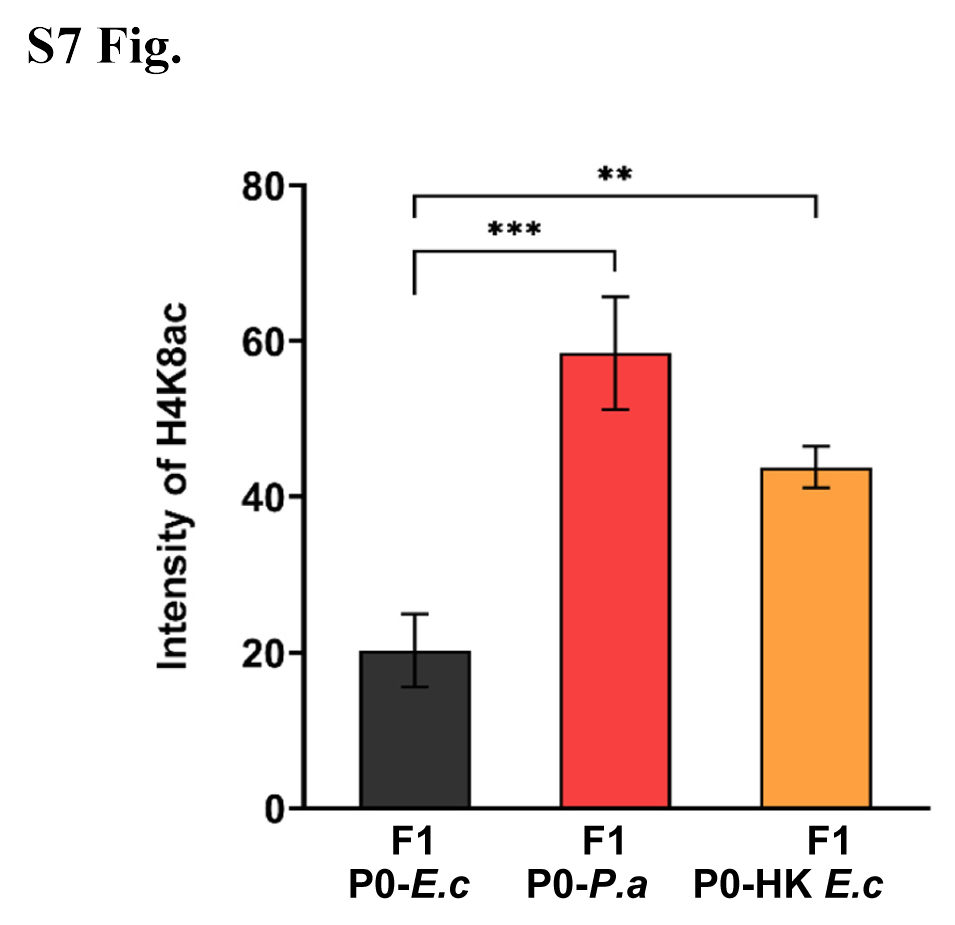

Supplement: S7 Fig — Quantification of immunofluorescence of H4K8ac levels on F1 progeny from P0 maternal animals exposed to E. coli (E. c), P. aeruginosa (P. a), or heat-killed E. coli (HK E. c) (n = 20). Three independent experiments were performed. “n” represents the number of animals for each experiment. “ns” indicates nonsignificant; “*” asterisk indicates significant difference; **P ≤ 0.005, ***P ≤ 0.0005. See S1 Data for the corresponding data. H4K8ac, histone H4 Lys8 acetylation. (TIF) [file pbio.3001169.s007.tif]
