# Supplementary material for: Microbial colonization induces histone acetylation critical for inherited gut-germline-neural signaling
Source: PLoS Biol. 2021 Mar 31;19(3):e3001169. doi: 10.1371/journal.pbio.3001169 (PMC8041202; doi:10.1371/journal.pbio.3001169)
Supplement: S1 Raw Images — (PDF) [file pbio.3001169.s008.pdf]

S1_Raw_images

Uncropped immunoblot images

Abbreviations: *'E. C'- E. coli, 'P. A'- Pseudomonas aeruginosa,* kD- kilo Dalton, *'X'- excluded sample/s,* vec- vector.

Fig 1A.

H4K8ac

Repeat 1

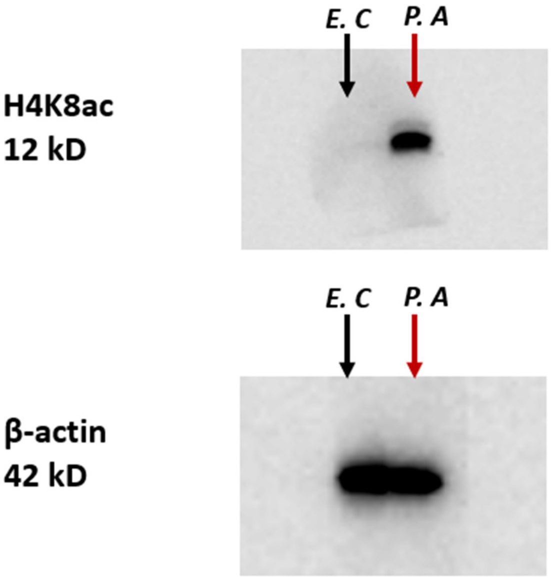

Repeat 2

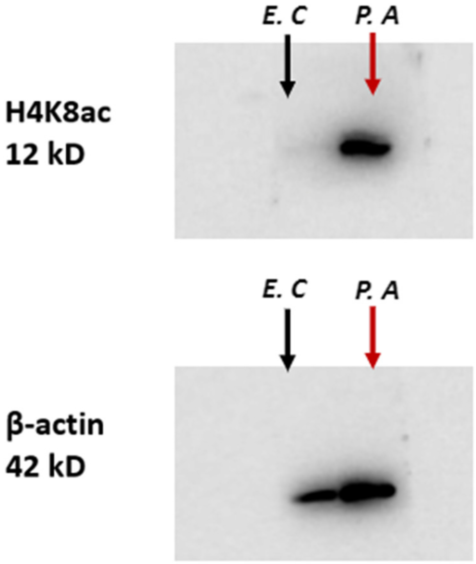

Repeat 3

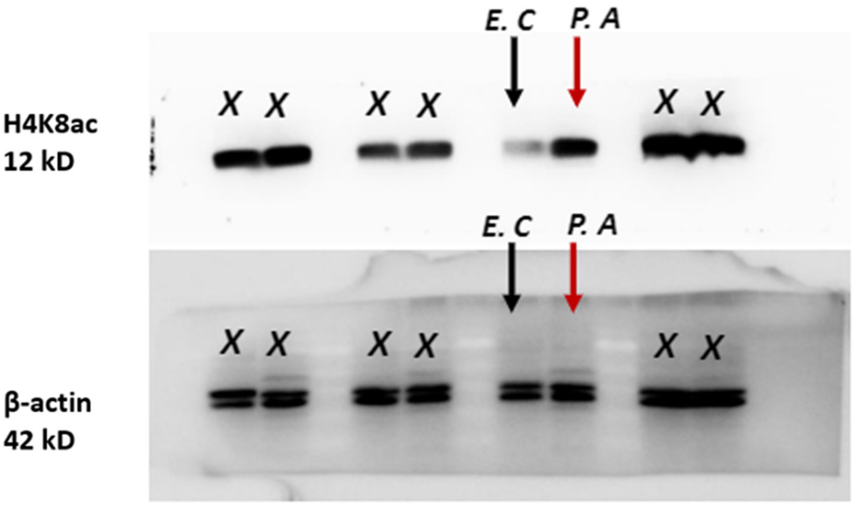

H3Kme1

Repeat 1

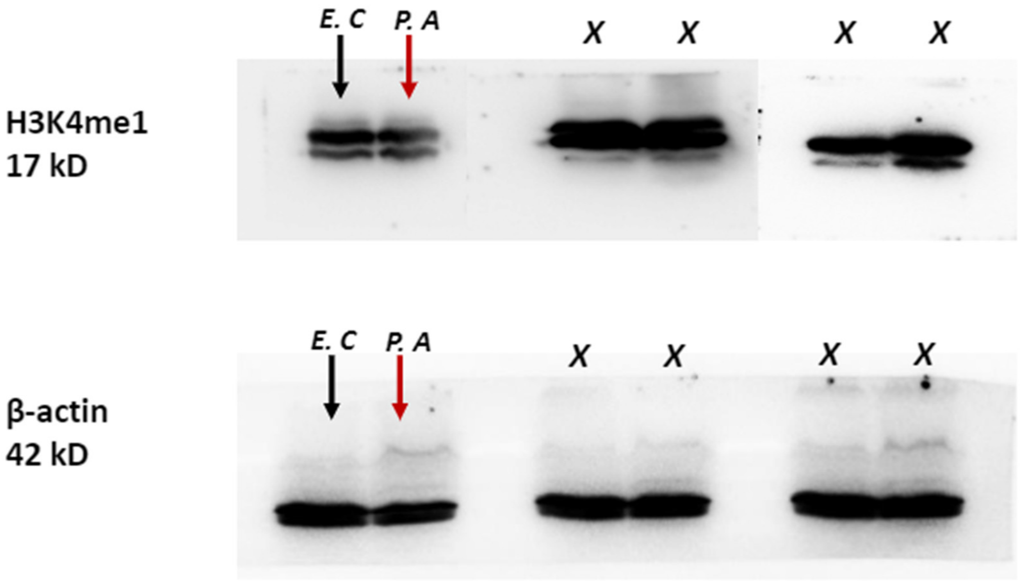

Repeat 2 and 3

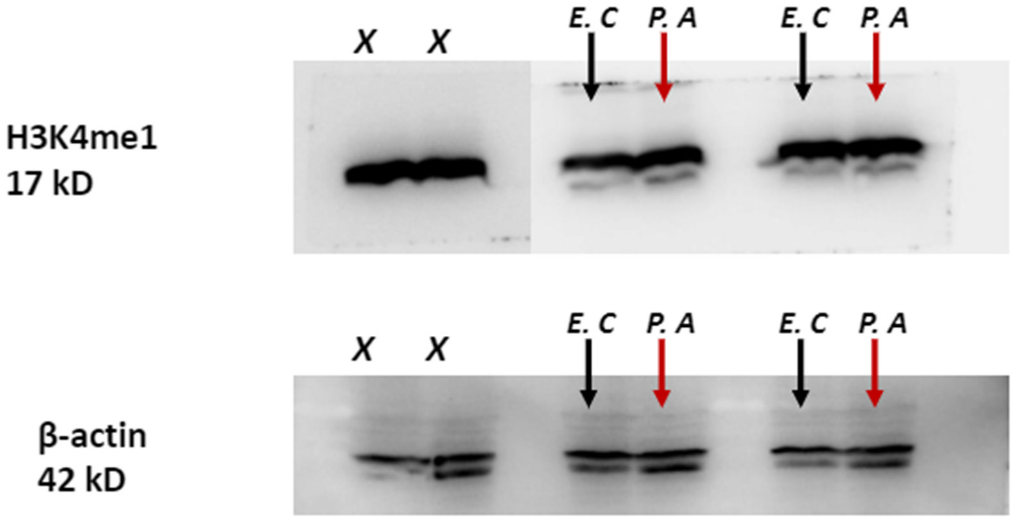

H3K4me3
Repeat 1

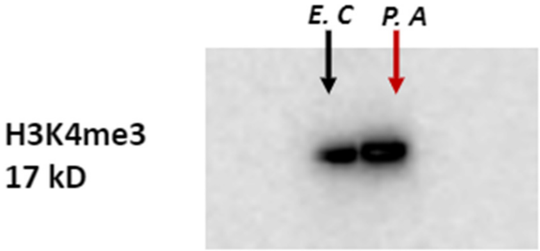

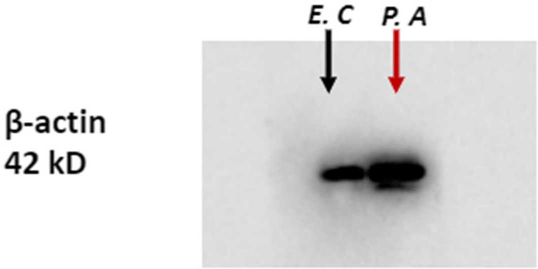

Repeat 2

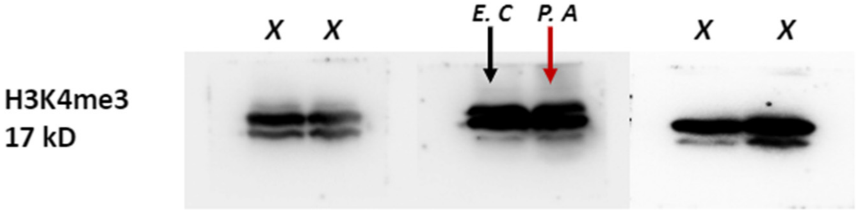

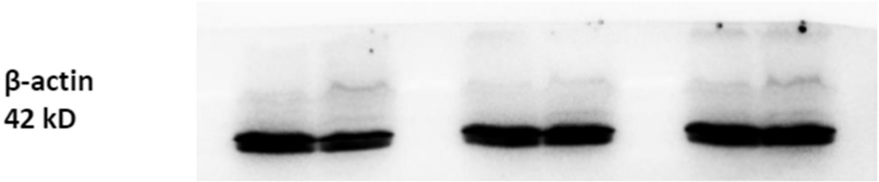

Fig. 1B

Repeat 1

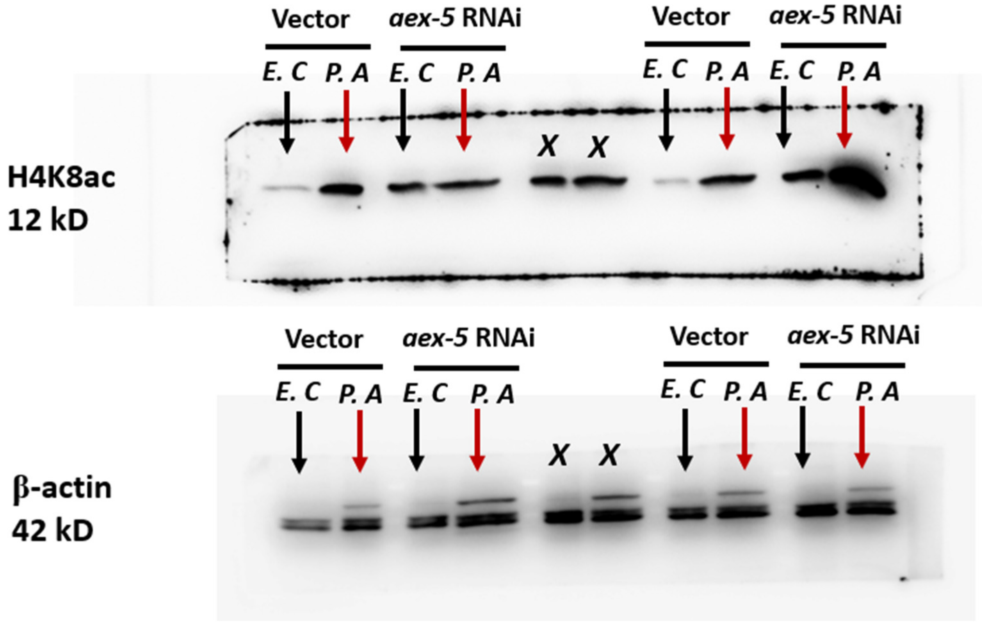

Repeat 2

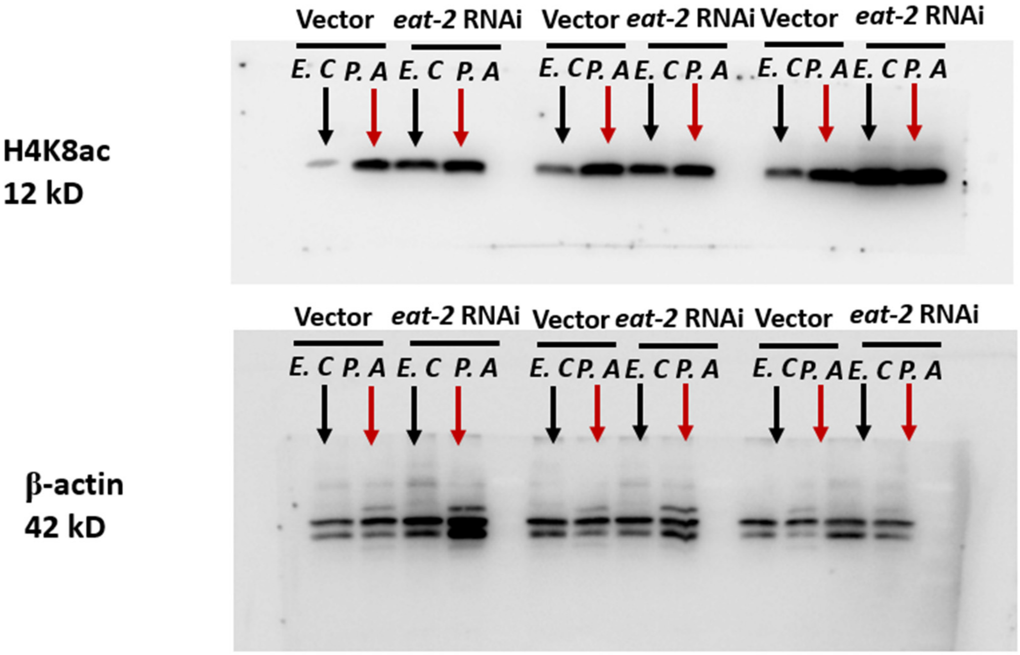

Repeat 3

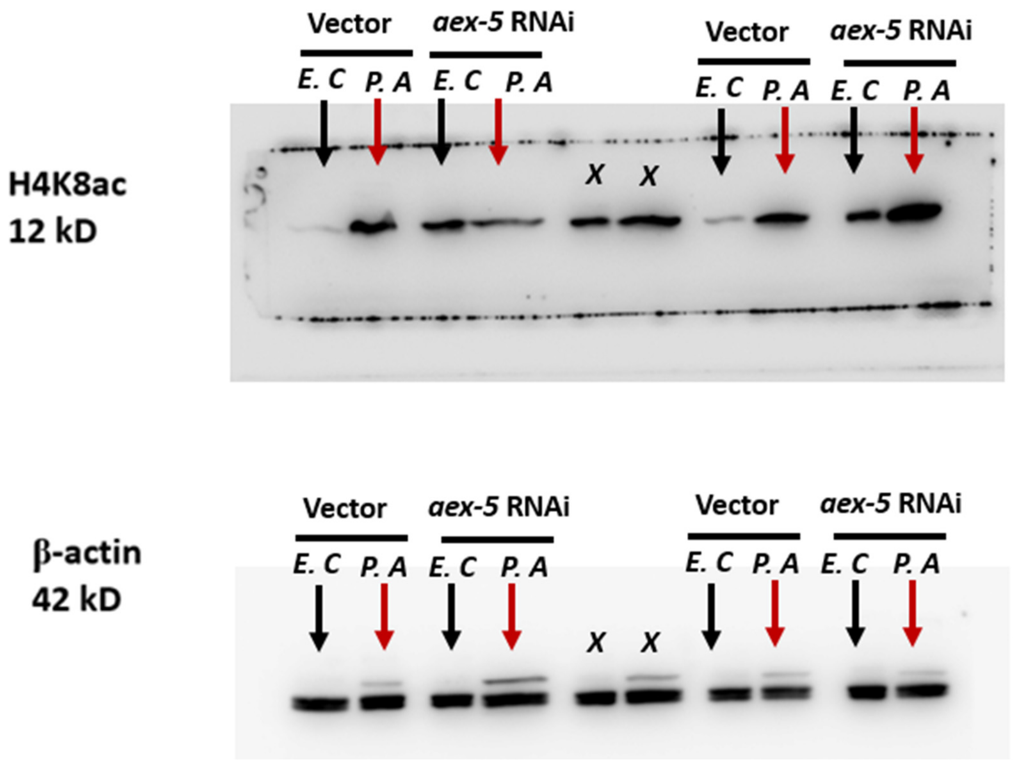

Fig 2A.
Repeat 1

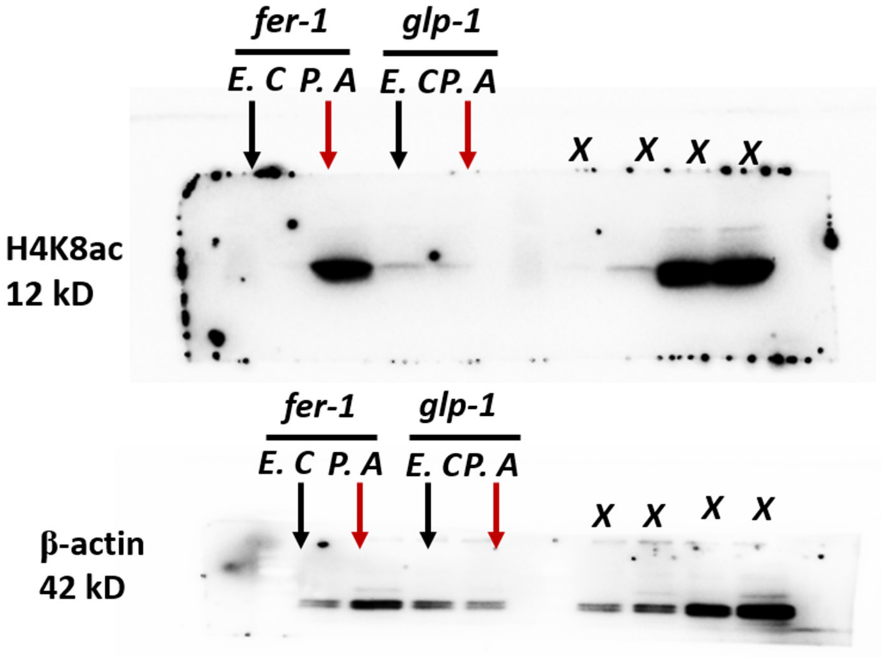

Repeat 2

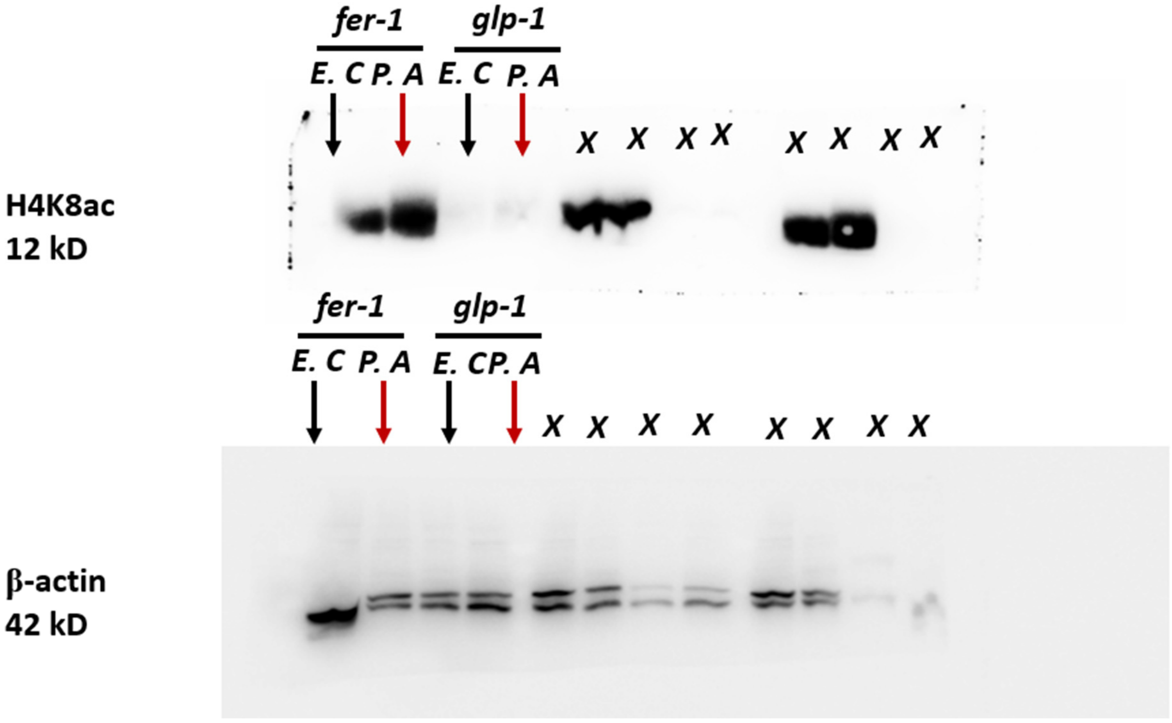

Repeat 3

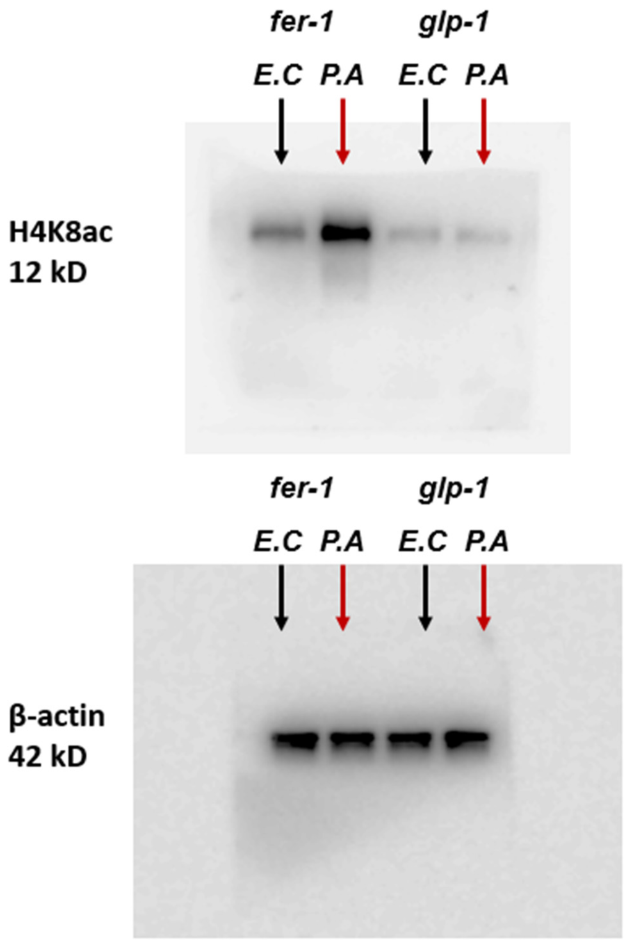
fer-1
glp-1
E.C
P.A
E.C
P.A
H4K8ac
12 kD
fer-1
glp-1
E.C
P.A
E.C
P.A
β-actin
42 kD

Repeat 4

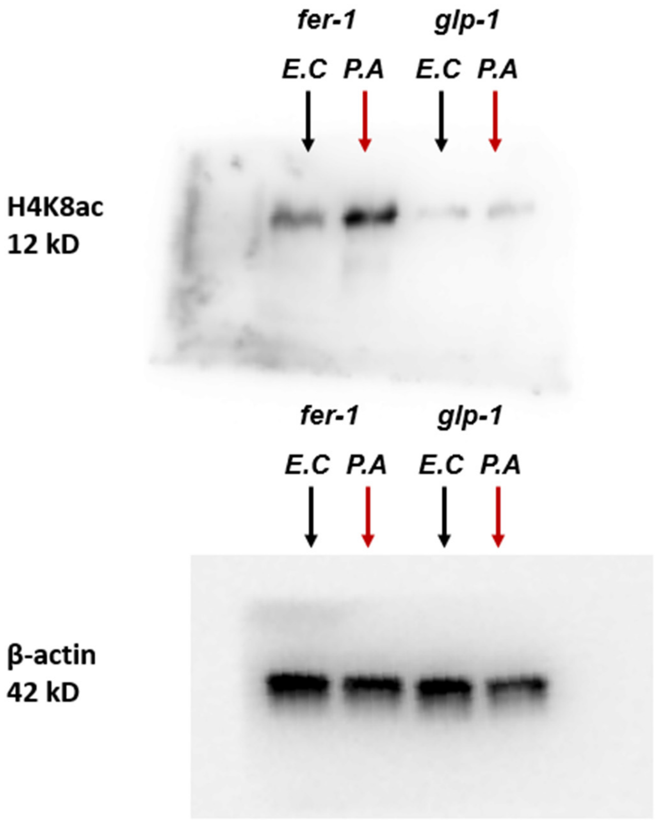

fer-1
glp-1
E.C
P.A
E.C
P.A
H4K8ac
12 kD
fer-1
glp-1
E.C
P.A
E.C
P.A
β-actin
42 kD

Fig. 3E

Repeat 1

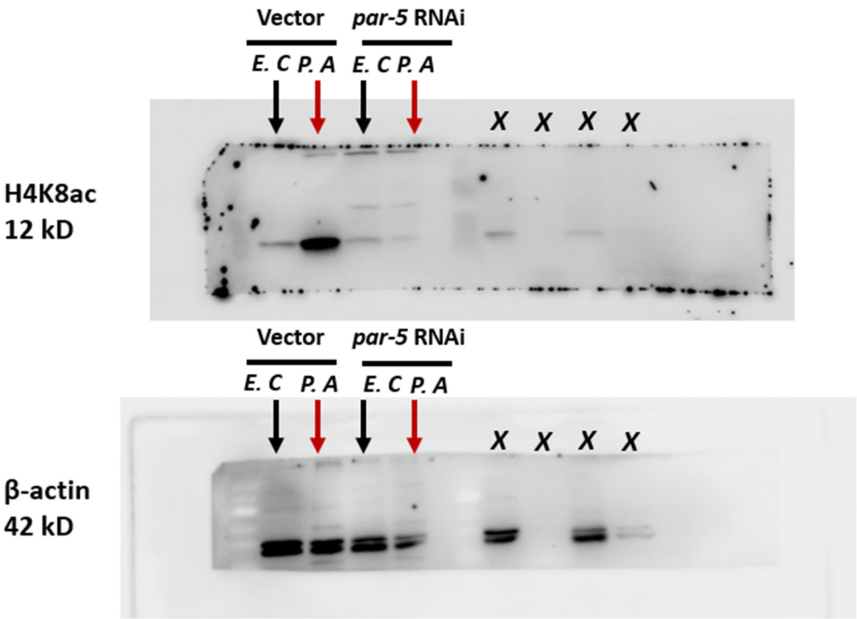

Repeat 2 and Repeat 3

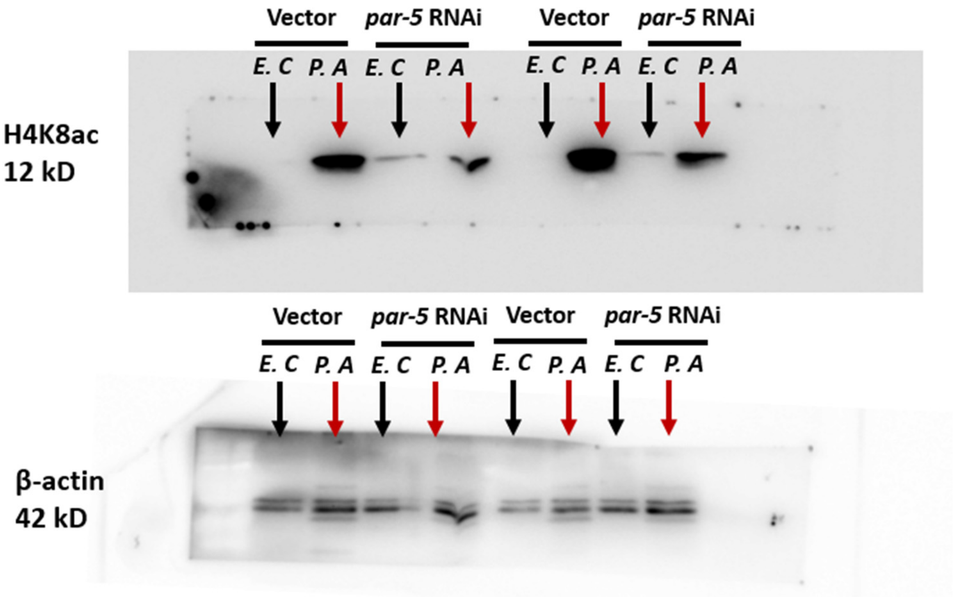

S3A Fig.

Repeat 1

PAR-5
28 kD

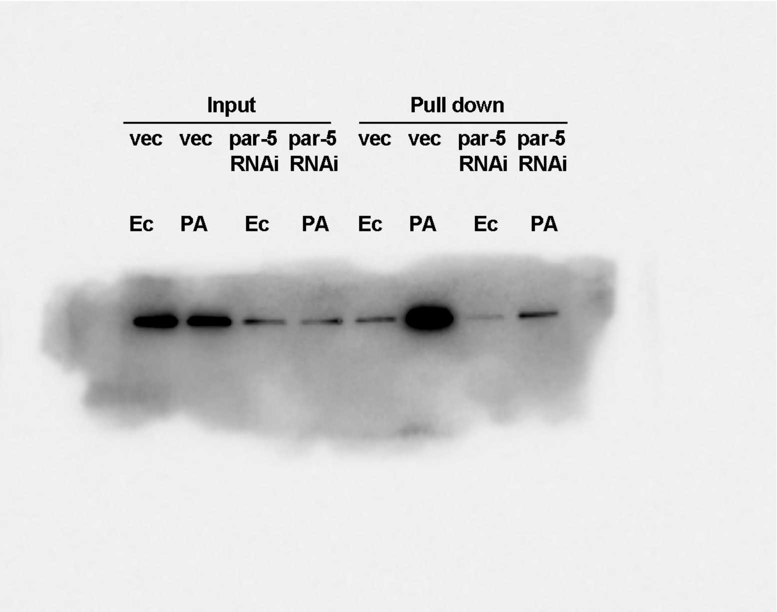

β-actin
42 kD

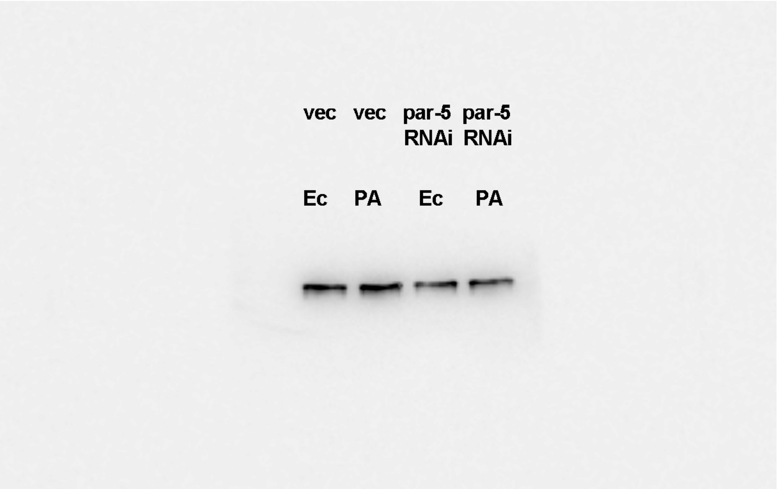

Repeat 2

PAR-5
28 kD

β-actin
42 kD

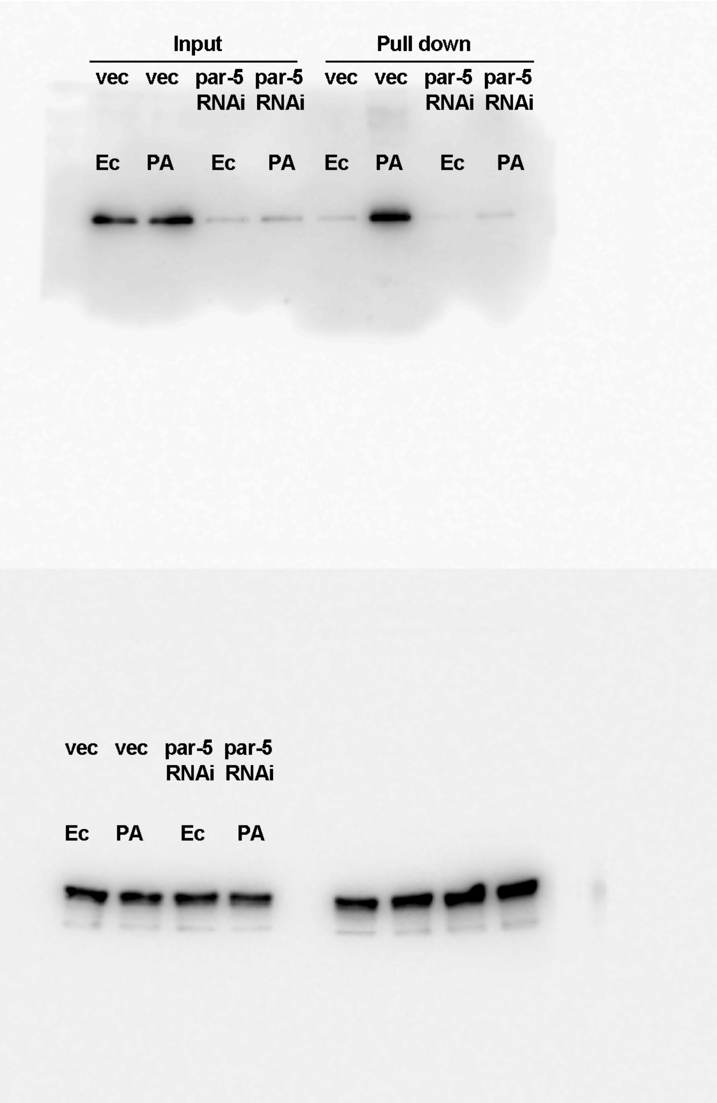

Repeat 3

PAR-5
28 kD

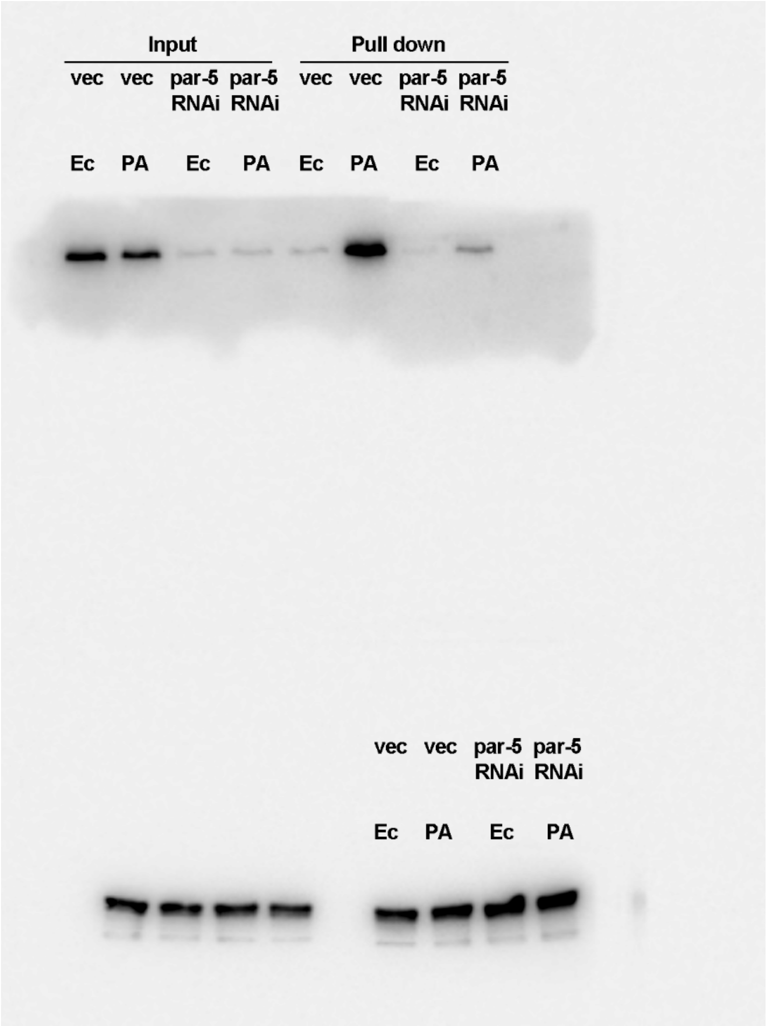

β-actin
42 kD

S4B Fig.

Repeat 1

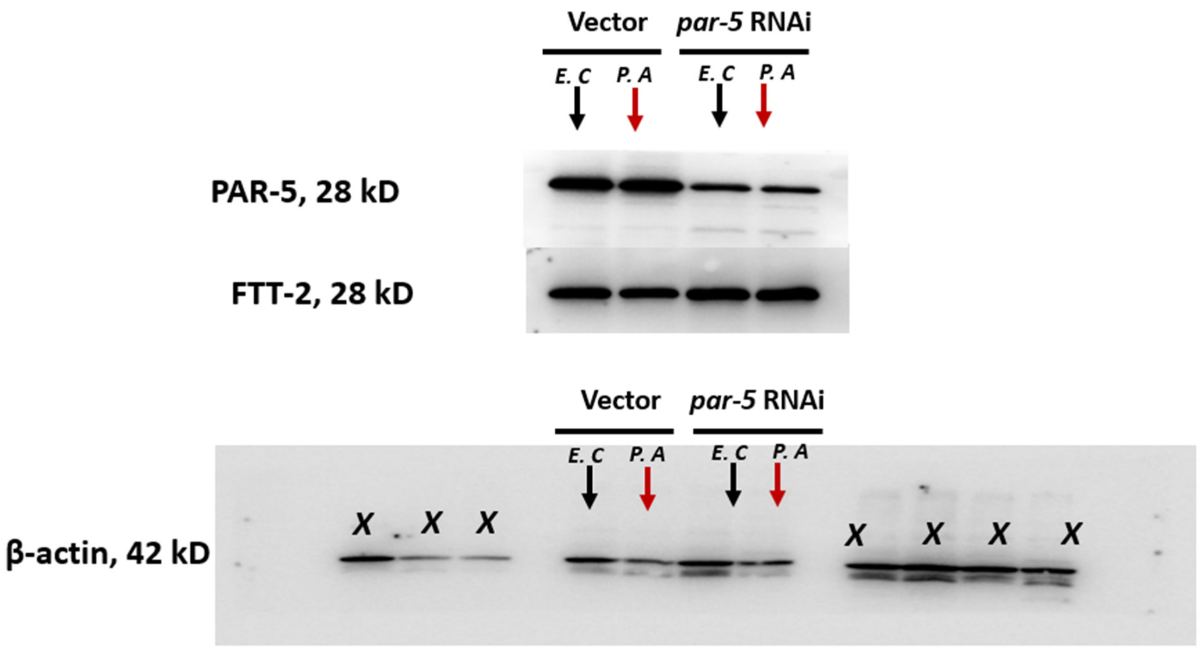

Repeat 2

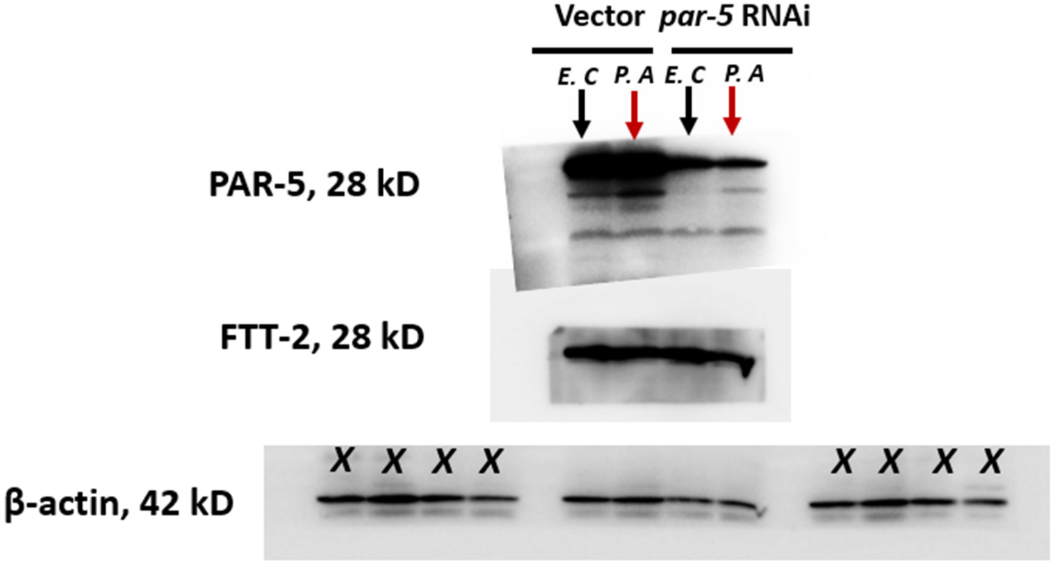

S5 Fig.

Repeat 1

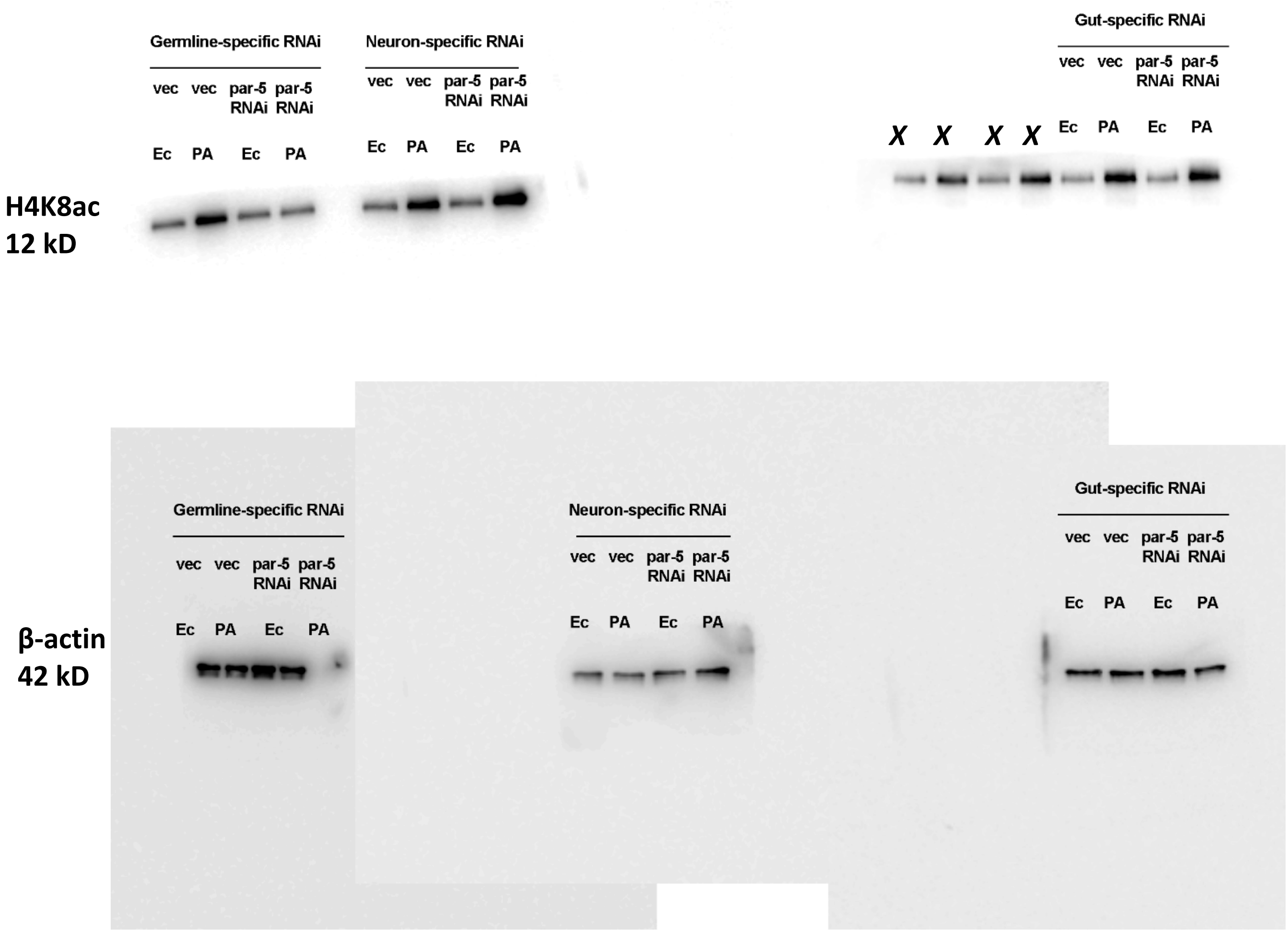

Repeat 2

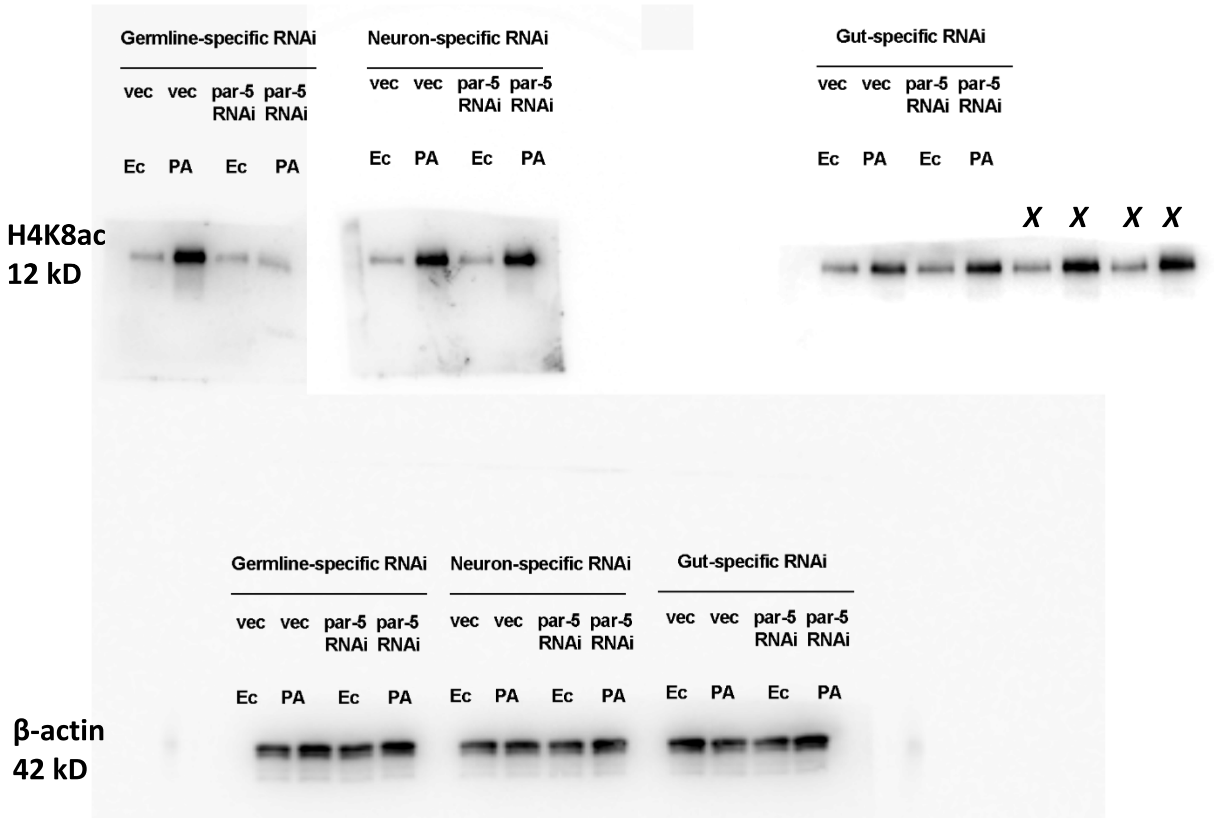
Germline-specific RNAi
Neuron-specific RNAi
Gut-specific RNAi
vec vec par-5 RNAi par-5 RNAi
Ec PA Ec PA
X X X X
H4K8ac
12 kD
Germline-specific RNAi
Neuron-specific RNAi
Gut-specific RNAi
vec vec par-5 RNAi par-5 RNAi
Ec PA Ec PA
β-actin
42 kD

Repeat 3

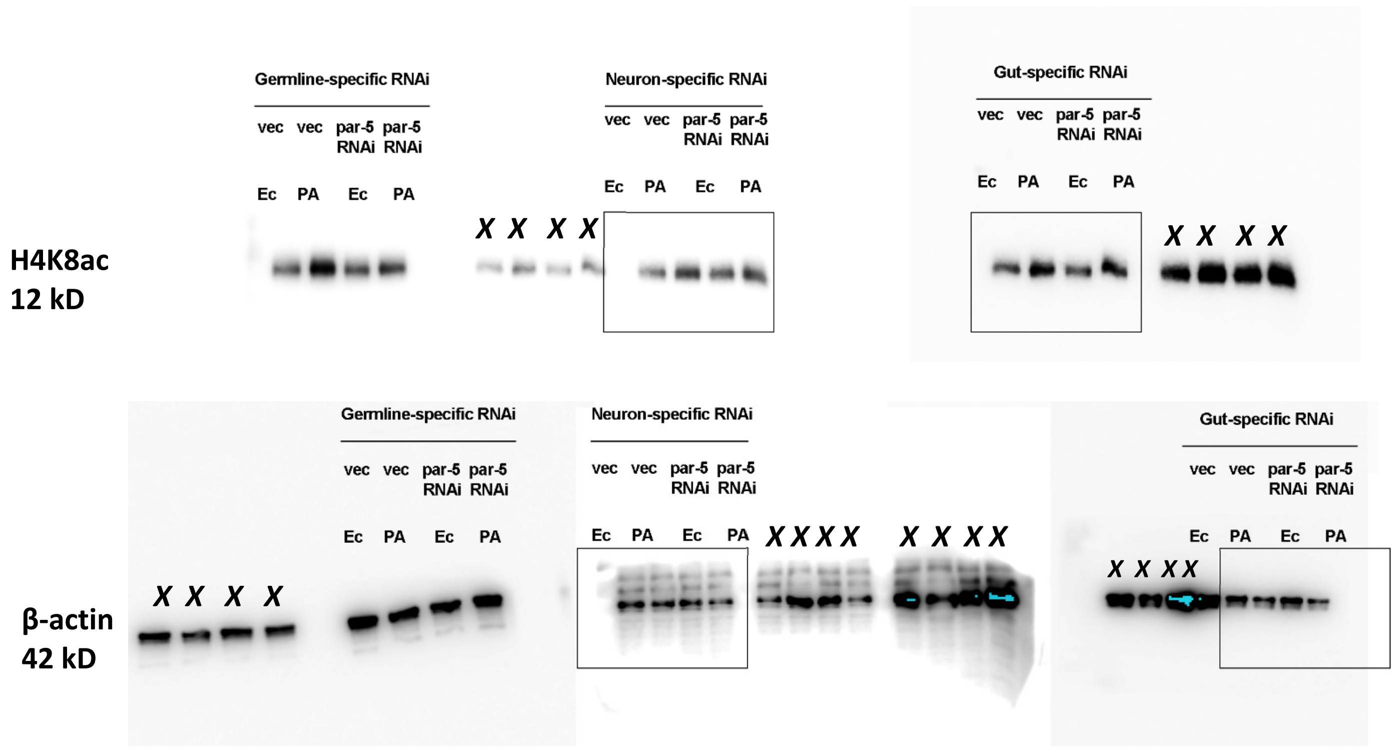
Germline-specific RNAi
vec vec par-5 RNAi par-5 RNAi
Ec PA Ec PA
Neuron-specific RNAi
vec vec par-5 RNAi par-5 RNAi
Ec PA Ec PA
Gut-specific RNAi
vec vec par-5 RNAi par-5 RNAi
Ec PA Ec PA
X X X X
X X X X
H4K8ac
12 kD
Germline-specific RNAi
vec vec par-5 RNAi par-5 RNAi
Ec PA Ec PA
Neuron-specific RNAi
vec vec par-5 RNAi par-5 RNAi
Ec PA Ec PA
Gut-specific RNAi
vec vec par-5 RNAi par-5 RNAi
Ec PA Ec PA
X X X X
X X X X
X X X X
X X X X
β-actin
42 kD
