# Supplementary material for: Microbial colonization induces histone acetylation critical for inherited gut-germline-neural signaling
Source: PLoS Biol. 2021 Mar 31;19(3):e3001169. doi: 10.1371/journal.pbio.3001169 (PMC8041202; doi:10.1371/journal.pbio.3001169)
Supplement: S1 Table — (PDF) [file pbio.3001169.s010.pdf]

S1 Table. List of H4K8ac interacting protein

WormBase Gene ID	Gene Name	*E. coli* (1)	*E. coli* (2)	Average (*E. coli*)	*P. aeruginosa* (1)	*P. aeruginosa* (2)	Average (*P. aeruginosa*)	Fold Change
WBGene00006921	vha-12	1	0	0.5	10	1	5.5	11.00
WBGene00013025	vha-13	2	0	1	9	7	8	8.00
WBGene00002007	hsp-3	2	0	1	12	2	7	7.00
WBGene00003514	myo-2	1	0	0.5	7	0	3.5	7.00
WBGene00020507	vha-15	1	0	0.5	4	2	3	6.00
WBGene00002008	hsp-4	2	0	1	9	2	5.5	5.50
WBGene00021355	Y37E3.17	2	0	1	8	2	5	5.00
WBGene00002000	hrp-2	0	1	0.5	4	1	2.5	5.00
WBGene00012553	cox-5A	1	0	0.5	5	0	2.5	5.00
WBGene00010317	idh-1	2	0	1	9	0	4.5	4.50
WBGene00003515	myo-3	2	0	1	8	0	4	4.00
WBGene00017166	aldo-2	1	0	0.5	4	0	2	4.00
WBGene00007350	sucl-1	1	0	0.5	4	0	2	4.00
WBGene00004441	rpl-27	1	0	0.5	3	1	2	4.00
WBGene00003920	par-5	1	1	1	5	2	3.5	3.50
WBGene00020950	dlst-1	1	1	1	3	4	3.5	3.50
WBGene00003162	mdh-2	8	3	5.5	26	11	18.5	3.36
WBGene00002083	inf-1	1	2	1.5	7	3	5	3.33
WBGene00004421	rpl-10	3	2	2.5	11	4	7.5	3.00
WBGene00002265	lec-2	1	1	1	4	2	3	3.00
WBGene00000991	dhs-28	2	0	1	4	2	3	3.00
WBGene00010965	ctc-2	1	0	0.5	3	0	1.5	3.00
WBGene00017864	pcca-1	1	0	0.5	3	0	1.5	3.00
WBGene00020366	acdh-10	1	0	0.5	3	0	1.5	3.00
WBGene00000230	atp-3	1	0	0.5	3	0	1.5	3.00
