# Supplementary material for: Microbial colonization induces histone acetylation critical for inherited gut-germline-neural signaling
Source: PLoS Biol. 2021 Mar 31;19(3):e3001169. doi: 10.1371/journal.pbio.3001169 (PMC8041202; doi:10.1371/journal.pbio.3001169)
Supplement: Attachment — Submitted filename: Response.pdf [file pbio.3001169.s012.pdf]

Response to all reviewers

We would like to thank the reviewers for their interest in our studies and their thorough review. Because Dr. Hong was not able to return to the lab due to the pandemic, we recruited several members of our laboratory to perform a number of experiments to substantiate our conclusions and experimentally address the reviewers' critiques. It was particularly challenging to perform additional experiments during lockdowns and strict social distancing rules that require us to work on shifts, but we believe that we have addressed the critiques by including new studies and making appropriate changes to the paper. The new studies we have added to the manuscript are shown in following new figures: Fig 1C, Fig 1D, Fig 2B, Fig 3A, Fig 3B, Fig 3C, Fig 3D, Fig 3F, S2C Fig, S2D Fig, S2E Fig, S3A Fig, S5 Fig, S6 Fig. Changes to the manuscript have been highlighted in gray.

We are grateful for the critiques, which have made the paper much stronger, and hope the reviewers find our responses satisfactory.

Reviewer #1:

COMMENT: *Hong and Aballay present a study that describes H4K8ac as an epigenetic mark in response to exposure to the pathogen PA14. They've shown that there is an increase in H4K8ac marks following pathogen exposure. The authors use a combination of genetics, RNAi, and biochemistry to define a germline response to PA14 with the ability to impact subsequent generations. In addition, the authors define additional players in the system to begin developing a pathway for this innate immunity response. While the data is presented clearly, there are some concerns with regard to interpretation of the results and the methodologies used that reduce enthusiasm for this short article. This potential importance of this finding and the need to identify additional mechanistic details suggest this should be a full-length article as in its present form many of the conclusions are correlative rather than causative.*

RESPONSE: We thank the reviewer for a very good summary. We have addressed all the deficiencies raised to strengthen the manuscript and the conclusions.

COMMENT: *1) The biggest drawback to this study is the lack of identification of the enzymes responsible for the addition of H4K8ac marks.*

RESPONSE: The enzymes involved in histone acetylation are known, and we respectfully believe that it is outside the scope of this work (that shows that *P. aeruginosa* infection induces H4K8ac, that the germline is required for the pathogen avoidance induced by intestinal bloating, and that the germline and PAR-5 are required for the transmission of pathogen avoidance to the next generation) to study H4 modifying enzymes in general.

As stated in the revised manuscript, we focused on PAR-5 because we observed a 3.5-fold increase in response to *P. aeruginosa* infection (S1 Table) and because it is expressed in both the nervous system and the germline. Our findings indicate that these two tissues play a role in the control of pathogen avoidance induced by pathogen colonization of the gut, which is the focus of this work. Thus, rather than focusing on known enzymes involved in H4K8 acetylation that also play major roles during the organismal growth and development, we focused on a partner that is involved in H4K8 acetylation during pathogen infection. The revised manuscript reads: "*We decided to further study PAR-5 because out of all the H4K8 acetylated-interacting candidate proteins that are upregulated more than 3-fold by P. aeruginosa infection, it is the only one that is expressed in the germline and in neurons, from where it could also be involved in the control of pathogen avoidance. Another reason why we focused on PAR-5 is that it belongs to a 14-3-3 family of chaperones [31, 32] that, through interactions with different proteins, can regulate PTM such as H4K8ac*"

The ChIP-MS assay suggests that during *P. aeruginosa* infection, PAR-5 is directly bound to histone. To further substantiate this result, we validated the binding using co-immunoprecipitation (New S3A Fig) and *in vivo* binding (New 3A, 3B, 3C, and 3D). Whole animal immunofluorescence IF staining also reveals that induction of H4K8ac by *P. aeruginosa* infection is dependent on *par-5* RNAi (New Fig 3F). Furthermore, new additional studies suggested by the reviewer (comment 2 below) strengthened the idea that PAR-5 controls the levels of H4K8 acetylation. More specifically, germline specific RNAi of *par-5* revealed not only disruption in pathogen avoidance but also H4K8ac (New S5 and New S6).

COMMENT: *2) Using glp-1 completely gets rid of germline so there could be confounding factors that affect pathogen avoidance, which are known. Why not use germline restricted RNAi or other germ cell manipulations (or gonad loss mutants) to strengthen this section? Although glp-1 mutants can indeed reduce the number of germ cells, they do not abolish all germ cells (it is incomplete).*

RESPONSE: It is standard in the field to use *glp-1* animals. Indeed, the work by Dr. Murphy that the reviewer mentioned linked the germline and pathogen avoidance using *glp-1* animals [Kaletsky, et al. "C. elegans interprets bacterial non-coding RNAs to learn pathogenic avoidance." Nature vol. 586,7829 (2020): 445-451. doi:10.1038/s41586-020-2699-5]. Our data indicate that *glp-1* animals, even though they have some residual germ cells, fail to avoid *Pseudomonas.* This indicates that the germ cells lacking in *glp-1* are required for pathogen avoidance. The manuscript reads: "*To study a potential role of the germline in gut-neural signaling, we used glp-1 animals, which lack most germline cells due to defects in mitotic and meiotic division [27, 28].*"

As requested by the reviewer, we have used germline restricted RNAi to confirm that PAR-5 functions in the germline to control pathogen avoidance (Fig. 3H). We have also included new additional experiments using tissue-specific RNAi strains, whole animal IF staining, and western blot assays to further validate the role of PAR-5 mediated acetylation of H48K in the germline (New S5 and New S6). The revised manuscript reads: "*Consistent with this idea, par-5 RNAi in the germline, but not in the intestine or in neurons, significantly suppressed the P. aeruginosa-induced H4K8ac (S5 Fig). Whole animal fluorescent immunohistochemistry confirmed that P. aeruginosa-induced H4K8ac is inhibited by par-5 RNAi in the germline (S6 Fig).*"

COMMENT: *Intriguingly, glp-1 mutants appear to have a low-level of H4K8ac (Figure 2A) with E. coli that does not increase with PA14?*

RESPONSE: We are not surprised to see H4K8ac in *glp-1* animals as it should not be restricted to the germline because histone acetylation is required for several physiological processes in different tissues. *fer-1* animals also have some level of basal H4K8ac that are not seen in Fig. 2A because of the differences in exposure and total amount of proteins. The quantification of the multiple experiments that takes into account the loading control (S2A Fig) shows this more clearly.

As noted by the reviewer, disruption of proper development of germline in *glp-1* animals resulted in lack of *P. aeruginosa*-induced H4K8ac, which is indeed the core of this study.

COMMENT: *3) The identification of PAR-5 is not clear. It was identified by ChIP-ms? More details are needed here as I assume this was done with the H4K8ac antibody, but it isn't clear why this would directly pull-down PAR-*

5. The authors should show that PAR-5 is binding directly to H4K8ac or define what it is interacting with. What is missing is a clear identification of what is PAR-5 regulating since it is a cytoplasmic chaperone. Since PAR-5 is important for embryo development and knockdown of par-5 could affect the germline in other ways as well the connection to H4K8ac is confounded.

RESPONSE: We hope the revised manuscript makes it clearer that PAR-5 was identified by ChIP-ms: "*To identify potential interacting partners that may affect H4K8ac in response to P. aeruginosa colonization, we performed chromatin immunoprecipitation-mass spectrometry (ChIP-MS). A total of 25 H4K8 acetylated-interacting candidate proteins that were upregulated more than 3-fold in infected animals were identified (S1 Table). We decided to further study PAR-5 because out of all the H4K8 acetylated-interacting candidate proteins that are upregulated more than 3-fold by P. aeruginosa infection, it is the only one that is expressed in the germline and in neurons, from where it could also be involved in the control of pathogen avoidance. Another reason why we focused on PAR-5 is that it belongs to a 14-3-3 family of chaperones [31, 32] that, through interactions with different proteins, can regulate PTM such as H4K8ac.*" The revised Method section mentions the anti-H4K8ac (ab15823, Abcam) antibody that was used to co-precipitate proteins that are bound to the specific histone. The details of cross-linking and precipitation are described in Methods.

As requested by the reviewer, we have performed additional co-immunoprecipitations that confirm the physical binding of PAR-5 with H4K8 (New S3A Fig). We also performed additional experiments to study potential nuclear interaction *in vivo*. We used bimolecular fluorescence complementation (BiFC) that is used to determine the physical interactions of proteins in living cells. The new study shows that there is *in vivo* physical interaction between PAR-5 and HIS-67, which is an ortholog of human H4 (New Fig 3A-D). Knockdown of *par-5* by RNAi resulted in the significant reduction of fluorescence (New Fig 3A-C), further confirming that the presence of the two proteins is required for the GFP reconstitution. The revised manuscript reads: "*We confirmed the direct binding of PAR-5 and H4 using co-immunoprecipitation (S3A Fig). We also confirmed the protein-protein interaction in vivo using bimolecular fluorescence complementation (BiFC), which allows for the determination of physical interactions of proteins in living cells through direct visualization [33]. The BiFC constructs were engineered to individually express, under the control of the heat shock promoter Phsp-16.41, GFP protein fragments translationally fused with PAR-5 and H4, which is a C. elegans ortholog of human H4. The interaction between the two proteins would bring the non-fluorescent fragments into close proximity for reconstitution and fluorescence. Twelve hours after heat shock, we observed fluorescence, indicating a physical interaction between PAR-5 and H4 in vivo (Fig 3A). Animals carrying BiFC constructs without H4 did not exhibit fluorescence. Knockdown of par-5 by RNAi resulted in a significant reduction of fluorescence (Fig 3B and 3C), further confirming that the presence of the two proteins is required for the GFP reconstitution. As shown in Fig 3A and 3D, the protein interaction occurs in the nuclei of hsp-16.41-expressing cells.*"

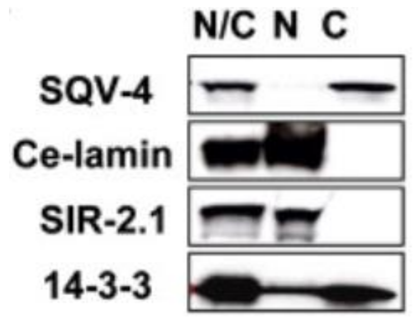

Fig 1E from Berdichevsky et al. Cell, 125, 6, P1165-1177, JUNE 13, 2006. To see whether 14-3-3 proteins are present in C. elegans nuclei, we performed cell fractionation experiments…14-3-3 proteins were present in both nuclear and cytosolic fractions (Figure 1E). These observations are consistent with the hypothesis that the interaction between SIR-2.1 and 14-3-3 proteins occurs in the nucleus.

We do not agree with the reviewer's statement that PAR-5 is a cytoplasmic chaperone. While the first line of *par-5* Overview in Wormbase (https://wormbase.org/species/c_elegans/gene/WBGene00003920#01e6-9gc4af3bd-10) reads "*par-5 (abnormal embryonic PARtitioning of cytoplasm)*," the same description reads "*Localizes to cell cortex and nucleus*." More importantly, published studies have shown PAR-5 nuclear localization (figure on the left). We agree that PAR-5 might affect H4K8ac indirectly, which should not detract from the many findings of this study (1-*P. aeruginosa* infection induces H4K8ac, 2-the germline is required for the

pathogen avoidance induced by intestinal bloating, and 3-intact germline and PAR-5 are required for the transmission of pathogen avoidance to the next generation). The new studies we performed to address the reviewer's critique shows PAR-5 nuclear localization and histone binding (New 3A-D, New S3A Fig). Additional studies provide further support to the role of PAR-5 on H4K8ac (3F, S5, and S6). Thus, as stated in the revised manuscript, we think that "*Even though PAR-5 is required for development and its inhibition may have wide effects on the germline that might indirectly affect H4 acetylation, our results indicate that PAR-5 directly interacts with histone.*"

COMMENT: *4) Based on the IF results, there appears to be some somatic cells with H4K8ac marks? Could the authors look at the levels of H4K8ac marks either through WB or IF for the tissue specific RNAi.*

RESPONSE: We performed the suggested experiments using both WB and IF. As shown in New S5 Fig and S6 Fig, only *par-5* RNAi in the germline significantly reduced H4K8ac. The revised manuscript reads: "*As shown in Fig 3H, par-5 RNAi in the germline significantly reduced pathogen avoidance, which is consistent with our previous results and suggests that H4K8ac occurs in the germline in response to infection. Consistent with this idea, par-5 RNAi in the germline, but not in the intestine or in neurons, significantly suppressed the P. aeruginosa-induced H4K8ac (S5 Fig). Whole animal fluorescent immunohistochemistry confirmed that P. aeruginosa-induced H4K8ac is inhibited by par-5 RNAi in the germline (S6 Fig).*"

COMMENT: *5) The connection to pathogen avoidance is the weakest as the germline has been shown to impact this response. The authors could look at H4K8ac levels in ftt-2 knockdown worms too since they share so much homology with PAR-5 but don't affect avoidance.*

RESPONSE: As the reviewer noted, the germline has been shown to impact pathogen avoidance. We do not have reason to believe that *ftt-2* knockdown will affect H4K8ac. Our studies show that *par-5* RNAi does not reduce the levels of FTT-2 (S4B and S4C Fig). The results of *ftt-2* knockdown would be difficult to interpret without studying its potential effect on *par-5* and the study of *ftt-2* is outside the scope of this work. The several aforementioned experiments we performed to address the previous criticism provide further support to the role of the germline in pathogen avoidance and H4K8ac (New Fig 3H, New S5 Fig, and New S6 Fig).

COMMENT: *6) Lastly, the authors should check what happens to H4K8ac marks following PA14 exposure if intestinal distension is suppressed like with nol-6 RNAi. If H4K8ac marks don't appear if intestinal distension is suppressed that would help back their claim.*

RESPONSE: We thank the reviewer for suggesting this experiment to substantiate our claim. As expected, *nol-6* RNAi animals suppresses the *P. aeruginosa*-induced H4K8ac (New S2D and S2E Fig).

COMMENT: *7) There are several temperature sensitive mutants used, but it is sometimes unclear what temperatures were used for raising animals in development, versus adulthood, versus experiment. Most seem to be done at 25 (with some exceptions), but the methods state that experiments were done at 20, unless otherwise indicated. It would be nice to have this explicitly outlined for each experiment.*

RESPONSE: We thank the reviewer for this suggestion. We have included the temperature and time conditions in each figure's legend.

COMMENT: *8) The authors include a whole section where they introduce PAR-5 and how 14-3-3 chaperones are involved with PTMs such as H4K8ac without citations.*

RESPONSE: We apologize for the oversight and we thank the reviewer for pointing out these insufficiencies. We have included relevant citation/s in the revised manuscript.

COMMENT: *9) Similarly, the transgenerational marks for H4K8ac are cool, but they should reference work that has already shown transgenerational inheritance of pathogen avoidance and specifically Coleen Murphy's work as possible future targets of study or H4K8ac inheritance.*

RESPONSE: We want to clarify that unlike colonization of the gut by live replicating *P. aeruginosa*, the exposure to RNA extracted from the pathogen does not induce intestinal bloating. This is not surprising as bloating requires intact, live replicating bacteria. Moreover, the RNA-induced avoidance accounts for only a small portion of the *P. aeruginosa*-elicited avoidance (25%, left figure).

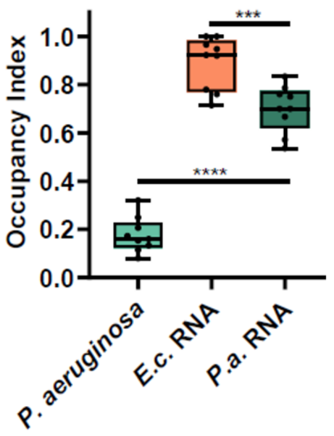

Occupancy index of N2 animals after 24-hour incubation on *P. aeruginosa* PA14 lawns or *E. coli* OP50 lawns supplemented with isolated total RNA from *E. coli* (*E.c.* RNA), *P. aeruginosa* (*P.a.* RNA), or *E. faecalis* (*E.f.* RNA).

We had an extensive discussion with Dr. Murphy, who agrees that our works are not sufficiently related and stated "*as opposed to our work on small RNA-induced learning and transgenerational inheritance of behavior, which seems orthogonal*." Dr. Murphy also stated the following: "*The focus of our paper is on the small RNA-driven part of the response*" (which as shown in the work by Murphy and the figure on the left, only accounts for a fraction of the total avoidance) "*I am also not sure what we would say about your study, since we also see no evidence of intestinal bloating in our experiments.*" Indeed, Dr. Murphy herself did not address our extensive work on bloating [Dev Cell. 2019 and Elife. 2019] in her study.

In deference of the reviewer's comment, we have discussed the work by the Murphy lab in the new Conclusions section: "*The inheritance of avoidance elicited by small RNAs from P. aeruginosa requires the germline [35, 36]. We do not know whether H4K8ac plays a role in the avoidance mediated by small RNAs, which accounts for a fraction of the avoidance elicited by P. aeruginosa.*"

Reviewer #2

COMMENT: *Although many bacterial infections are restricted to the intestine, there is increasing evidence that infection causes signaling between different tissues. C. elegans is a powerful system to study bacterial infections, pathogen avoidance, and effects that are passed down between generations. In this manuscript the authors investigate the connection between the intestine and germline, and how this affects animal behavior. The authors find that infection with Pseudomonas aeruginosa induces H4Kac methylation in the germline. This methylation also occurs by knocking down several genes that cause intestinal distension. These RNAi conditions also induce pathogen avoidance which is dependent on the germline. The authors also identify PAR-5 as interacting with*

H4Kac. The authors show that PAR-5 is necessary for H4Kac methylation in the germline and for bacterial avoidance. Finally, the authors show that under conditions that induce this methylation, that it can be passed on to the next generation and the progeny have increased bacterial avoidance. This is a very interesting and well-carried out study that provides new insight into the connection between the intestine and germline and how communication between these tissues can influence behavior.

RESPONSE: We thank the reviewer for a very good summary and support to our study.

COMMENT: *1) The authors claim that "results demonstrate that enhanced H4K8ac in the germline is required for the transgenerational pathogen avoidance induced by bloating caused by bacterial colonization of the intestine" and claim in 4c that PAR-5 is required for this transgenerational effect. The authors do not show that animals that lack H4K8ac in the germline generate progeny that are defective for bacterial avoidance. They also don't show that PAR-5 is necessary for this transgenerational effect. Although this experiment is not possible with par-5 RNAi, the authors should either conditionally deplete PAR-5 (such as with auxin inducible degradation) in the P0s, or reword the text and figure to remove these claims.*

RESPONSE: The reviewer raised a good point about the limitation of our study. As suggested, we have removed the claims and used the term 'intergenerational' instead of 'transgenerational.'

COMMENT: *2) The convention in the field is to only use "transgenerational" to refer to effects that are passed down at least three generations (Perez and Lehner nature cell biology 2019). Effects that are only shown to be passed down a single generation are referred to as "intergenerational". Although the effect shown in this manuscript may indeed be transgenerational, the authors have not shown this. The authors should either test how many generations this effect lasts or change the text to clarify that it may be either intergenerational or transgenerational.*

RESPONSE: Thank you again for highlighting this issue. We have used the term "intergenerational" in the revised manuscript.

COMMENT: *3) Insert in the following sentence "not" after "did": "As shown in Fig 2B, inhibition of aex-5 and eat-2 did elicit pathogen avoidance in glp-1 animals."*

RESPONSE: We apologize for this error that has been corrected.

COMMENT: *4) In Figure 1C and 3A, outlines of the germline are necessary as it is hard to know where the germline is in the current fluorescent images.*

RESPONSE: To address the reviewer's critique, we performed additional experiments and took better quality images. Also, we included close-up images of the germline region and included outlines (new Fig 1C). We have included differential interference contrast (DIC) representative images (new Fig 1C) and also new images that depict the entire body of the animal (new Fig 1D). We replaced the images in Fig 3A with better quality images from the new experiments (new Fig 3F). We decided to apply outlines only to the close-up images because we believe the outlines on other figures reduce the overall quality (below).

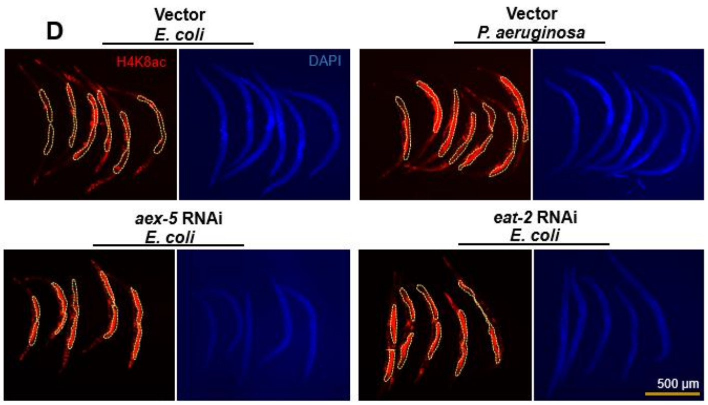

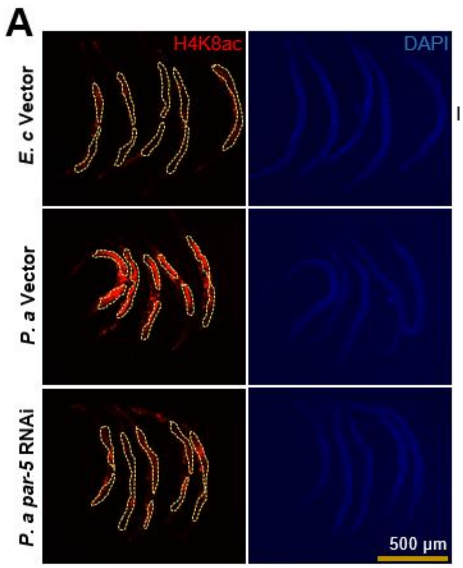

COMMENT: *5) Other examples of pathogen avoidance being transmitted to progeny have been demonstrated in C. elegans and should be cited:*

RESPONSE: We have discussed the work indicating that small RNAs induce transgenerational inheritance of pathogen avoidance: "*The inheritance of avoidance elicited by small RNAs from P. aeruginosa requires the germline [35, 36]. We do not know whether H4K8ac plays a role in the avoidance mediated by small RNAs, which accounts for a fraction of the avoidance elicited by P. aeruginosa.*"

COMMENT: *6) There are several instances of "C elegans" which should be "C. elegans".*

RESPONSE: We apologize for these errors. Necessary corrections were made in the revised manuscript.

Reviewer #3

COMMENT: *The work of Hong and Aballay aims at contributing to the elucidation of the mechanisms involved in the determination of behavior by signaling from the gut. The particular question the group is addressing is how the intestinal distention caused by bacteria P. aeruginosa in C. elegans mediates avoidance behaviors (by measuring the permanence of animals in the pathogen's lawn). The authors find that the acetylation of lysine 8 in histone 4 in the gonad is caused by intestinal distention and is essential for both behavioral avoidance and inheritance. Authors highlight a role for the gonad in the intestinal-brain communication axis that triggers behavioral change. This is a fascinating topic of great significance for the field.*

RESPONSE: We thank the reviewer for a very good summary and support to our study.

COMMENT: *1) There is no mention in the text of the role of histone modification and specifically of what the H4K8ac is doing transcriptionally. Also, authors do not discuss why this specific modification could be occurring compared to H3K4me3 or H3K4me1, and why were these three selected.*

RESPONSE: We have included more information regarding the role of histone modification on gene expression and provided a better rationale for selecting the three modifications studied. The revised manuscript reads: "*Histone post-translational modifications (PTM) are the most common epigenetic mechanisms, and different modifications have been found to be involved in diverse biological processes across species, including C. elegans [18- 20]. Methylation and acetylation are common histone PTM that generally affect gene expression by altering the activity of origins of DNA replication or chromatin structure and gene transcription [21, 22]. As a first step to studying whether histone PTM play a role in the control of the pathogen-aversive behavior elicited by microbial colonization of the C. elegans intestine, we looked at mono-methylation of histone H3 Lys4 (H3K4me1), tri-methylation of histone H3 Lys4 (H3K4me3), and histone H4K8ac as they have been linked to immunological memory in plants and mammals [23, 24].*"

COMMENT: *2) Authors used a t-test for their analysis (as mentioned in the methods section). They should instead use a one-way ANOVA with post-hoc analysis for those experiments that contain more than two conditions.*

RESPONSE: As suggested by the reviewer, we have re-analyzed the studies that require ANOVA and post-hoc statistics.

The revised manuscript reads: "*Two-tailed Student's t-test for independent samples was used to analyze the data. For comparing the means of more than two groups, one-way ANOVA with post-hoc analysis was performed. All the experiments were repeated at least 3 times and error bars represent the standard deviation, unless otherwise indicated. The data was judged to be statistically significant when $P < 0.05$.*"

COMMENT: *3) Figure 1C. It is really hard to see the gonad in these pictures or distinguish it from any other structure. A bright field or Nomarski picture should be provided and a marker to confirm the localization of the marks. It would be important to show whether neurons are also marked. Can authors explain why animals appear curved?*

RESPONSE: We performed new experiments and replaced the figures. We have included close-up images of the germline acetylation and Nomarski images to improve visualization (new Fig 1C). We have included new images showing the entire body of the animals (new Fig 1D). We also replaced the images in Fig 3A with better quality images from new experiments (new Fig 3F).

We believe that some animals are curved due to the desiccation caused by fixation and permeabilization needed for the antibody treatment.

COMMENT: *4) As in point 3, images in Figure S5 need improving. It is hard to distinguish the germline in the photos. Also, there is expression (red color) elsewhere. Which cells are those? It is important that authors show Nomarski images for those staining's. It will really help to show an independent marker for the gonad or provide clear DAPI images.*

RESPONSE: As requested, we have included improved Nomarski and DAPI images (New Fig 2B, which was former figure S5).

COMMENT: *5) Figure legends need more detail. For example: Quantification of western blots of extracts from fer-1(b232) animals exposed to E. coli (E. C) or P. aeruginosa (P. A). What is it that is being quantified? Fold change of what? How is this calculated? How many animals in each experiment? In the same line, this should be clarified in the figure axis (applies to all graphs with fold change). For those graphs quantifying intensity of histone acetylation, the same clarification will be important. Are those pixels? Intensity over a control?*

RESPONSE: We have included the requested information in the figure legends (the changes are highlighted in gray).

As better explained in both the legends and materials and methods, fold change corresponds to the mean density of a given sample over the control *E. coli* sample. β-actin was used as an internal reference control to normalize the values of each group. For the quantification of fluorescence images, we used ImageJ and the whole animal fluorescence was calculated using the following equation: corrected whole animal fluorescence = integrated density – (area of selected animal x mean fluorescence of background readings).

COMMENT: *6) In the second paragraph of the second section of results reads "As shown in Fig 2B, inhibition of aex-5 and eat-2 did elicit pathogen avoidance in glp-1 animals". What figure 2B shows is the opposite. I imagine this is a typo (an important one) given what is said afterward.*

RESPONSE: We apologize for this typographical error that was corrected. The revised manuscript reads (new 2C, which was former Fig 2B): "*As shown in Fig 2C, inhibition of aex-5 and eat-2 did not elicit pathogen avoidance in glp-1 animals.*"

COMMENT: *7) Figure 2C will be very hard to understand for a general audience. I suggest (as mentioned before for the other images) to include a Nomarski image. In these pictures, the glp-1 mutants do not appear equally colonized by PA14-GFP as wild type animals. Additionally, there are other methods that more accurately measure the number of bacteria colonizing the animal intestine (CFU count for instance).*

RESPONSE: As per reviewer's suggestion, we have performed additional CFU count to determine the extent of *P. aeruginosa* colonization (new S2C Fig). We also improved the quality of the images (modified Fig 2D, which was former Fig 2C).

COMMENT: *8) The experimental paradigm used in this work does not correspond to transgenerationally inherited phenomena. For an effect to be transgenerational animals should skip at least one generation of encounter to the pathogen and the following generation examined (see Rechavi's papers for examples of transgenerational paradigms. For transgenerational effects involving pathogens see Palominos et al., 2017 or Moore et al., 2019). The effect observed here could be called intergenerational instead.*

RESPONSE: Thank you for bringing this up. As requested, we have used the term "intergenerational" instead.

COMMENT: *9) In the quantifications of immunofluorescence (Supplementary Figures) the n values seem to be random. For example: S6 Fig n=4; S8 Fig. n=5; S11 Fig n=20. Are these n=4, n=5, n=20 per condition? 4, 5 or 20 on each bacterium or RNAi experiment? Or is it the total of animals screened. In any case, why are the numbers so different?*

RESPONSE: Overall, the number of animals used in avoidance assays is much larger than that used in microscopy. We have made it clearer in the legends that the numbers correspond to the total number of animals screened and the number of each independent experiment.

COMMENT: *10) Other points (or simple suggestions):*
I suggest to mention throughout the text the strain of P. aeruginosa used is PA14.

RESPONSE: As a common rule, we use *P. aeruginosa* in our manuscripts because we believe it is better for the general audience to have the pathogen name rather than the strain name. We also use *C. elegans* instead of N2. We used the abbreviation "*P.a*" in some figures because it is shorter than PA14 and thus allows us to use larger fonts.

COMMENT: *In the first paragraph of the Results section authors state "P. aeruginosa colonization specifically induces histone H4K8 acetylation in the infected animals". In the following paragraph it is said that distention alone causes H4K8 acetylation. This apparent contradiction could be avoided by rephrasing the first sentence with "colonization" or other similar word because at that point they do not know whether it is specific to the pathogen.*

RESPONSE: We thank the reviewer for noting this issue. The revised manuscript reads: "*We found that only H4K8ac increased (Fig 1A and S1A Fig), suggesting that P. aeruginosa infection induces histone H4K8 acetylation in the infected animals.*
Because infection by P. aeruginosa correlates with colonization and distension of the C. elegans intestinal lumen, which triggers bacterial aversion [16, 26], we reasoned that H4K8ac may also increase by intestinal distension."

COMMENT: *It would be nice if all graphs had a homogenous font size.*

RESPONSE: As per the reviewer's suggestion, we re-drew the graphs and used uniform font sizes whenever possible.

In summary, we would like to thank the reviewers one more time for providing constructive critiques. We believe we have fully addressed all the concerns of the reviewers by carrying out several new experiments. The manuscript has undergone a substantial revision, and thereby improved significantly. We do hope that the revised manuscript will be found suitable for publication in *PLOS Biology*.
