# Supplementary material for: Microbial colonization induces histone acetylation critical for inherited gut-germline-neural signaling
Source: PLoS Biol. 2021 Mar 31;19(3):e3001169. doi: 10.1371/journal.pbio.3001169 (PMC8041202; doi:10.1371/journal.pbio.3001169)
Supplement: Attachment — Submitted filename: Response.pdf [file pbio.3001169.s013.pdf]

Response

We would like to thank the Editor and staff members for processing and handling our manuscript submission. Please find below the responses to all the requests.

EDITORIAL REQUEST: *If Supporting Information files are included with your article, note that these are not copyedited and will be published as they are submitted. Please ensure that these files are legible and of high quality (at least 300 dpi) in an easily accessible file format. For this reason, please be aware that any references listed in an SI file will not be indexed.*

RESPONSE: We have ensured our Supporting information files are legible and the figures are of high quality.

EDITORIAL REQUEST: **Protocols deposition**
To enhance the reproducibility of your results, we recommend that if applicable you deposit your laboratory protocols in protocols.io, where a protocol can be assigned its own identifier (DOI) such that it can be cited independently in the future.

RESPONSE: Details of our protocols are available within the manuscript.

EDITORIAL REQUEST: *DATA POLICY: Note that we do not require all raw data. Rather, we ask that all individual quantitative observations that underlie the data summarized in the figures and results of your paper be made available in one of the following forms:*

1) Supplementary files (e.g., excel). Please ensure that all data files are uploaded as 'Supporting Information' and are invariably referred to (in the manuscript, figure legends, and the Description field when uploading your files) using the following format verbatim: S1 Data, S2 Data, etc. Multiple panels of a single or even several figures can be included as multiple sheets in one excel file that is saved using exactly the following convention: S1_Data.xlsx (using an underscore).

REPONSE: We have created Supplementary files as recommended. We have compiled a single excel sheet file (S1_Data) that has multiple tabs; each tab contains quantitative data for our observations and results.

EDITORIAL REQUEST: *2) Deposition in a publicly available repository. Please also provide the accession code or a reviewer link so that we may view your data before publication.*

REPONSE: Not applicable.

EDITORIAL REQUEST: *Regardless of the method selected, please ensure that you provide the individual numerical values that underlie the summary data displayed in the following figure panels as they are essential for readers to assess your analysis and to reproduce it:*

Fig. 2C, E; Fig. 3B, C, G, H; Fig. 4B; Fig. S1A-D; Fig. S2A-C, E; Fig. S3A-C; Fig. S4A, C; Fig. S5; Fig. S6B and Fig. S7

NOTE: the numerical data provided should include all replicates AND the way in which the plotted mean and errors were derived (it should not present only the mean/average values).

REPONSE: As recommended, we have generated a supporting data file that contains underlying data for the figures Fig. 2C, E; Fig. 3B, C, G, H; Fig. 4B; Fig. S1A-D; Fig. S2A-C, E; Fig. S3A-C; Fig. S4A, C; Fig. S5; Fig. S6B and Fig. S7. We have compiled a single excel sheet file (S1_Data) that has multiple tabs; each tab containing numerical values from independent assays, plotted means and errors.

EDITORIAL REQUEST: *Please also ensure that figure legends in your manuscript include information on WHERE THE UNDERLYING DATA CAN BE FOUND, and ensure your supplemental data file/s has a legend.*

Please ensure that your Data Statement in the submission system accurately describes where your data can be found.

REPONSE: As recommended, we have revised the figure legends in the manuscript. For example,
"See S1_Raw_images for uncropped immunoblot images and S1_Data for the underlying data."

EDITORIAL REQUEST: *BLOT AND GEL REPORTING REQUIREMENTS:*

For manuscripts submitted on or after 1st July 2019, we require the original, uncropped and minimally adjusted images supporting all blot and gel results reported in an article's figures or Supporting Information files. We will require these files before a manuscript can be accepted so please prepare and upload them now.

RESPONSE: As recommended, we have generated a supporting Raw image file (S1_Raw_images) that contains all the uncropped immunoblots images.

Additional corrections:

- S1D Fig. X-axis label corrected. "*P.a" eat-2* RNAi is corrected as "*E.c*" *eat-2* RNAi.
- S2A Fig, S2E Fig, S3B, S5 Fig, and S6B Fig, graphs are re-plotted to include data from more independent experiments. Figure legends are also revised accordingly.
- Figure legends are revised as per the recommendation (to inform readers of the location of the underlying data and RAW images). For example,

  "See S1_Raw_images for uncropped immunoblot images and S1_Data for the underlying data."
