# Supplementary material for: Microbial colonization induces histone acetylation critical for inherited gut-germline-neural signaling
Source: PLoS Biol. 2021 Mar 31;19(3):e3001169. doi: 10.1371/journal.pbio.3001169 (PMC8041202; doi:10.1371/journal.pbio.3001169)
Supplement: Attachment — Submitted filename: Response.pdf [file pbio.3001169.s014.pdf]

Response

EDITORIAL REQUEST 1: *Figure S1C: Values in independent assays 1 and 2 are exactly the same. Are these really independent or is it a mistake? If so, please correct it and add the right values.*

RESPONSE: Thank you for noticing this mistake. We have updated the “S1_DATA” file with the correct numbers that correspond to three western blots.

EDITORIAL REQUEST 2: *Figure S4C. There are only 2 replicas – could you please explain why? Please note that we do require 3 replicates for all experiments.*

RESPONSE: Figure S4C corresponds to experiments we performed to set up the RNAi technology. We do not believe it is necessary to repeat those very clear westerns that show that *par-5* RNAi downregulates the amount of PAR-5 protein (left, below). We have functionally showed the specificity of *par-5* RNAi in 3 independent experiments (S4A)(right, below).

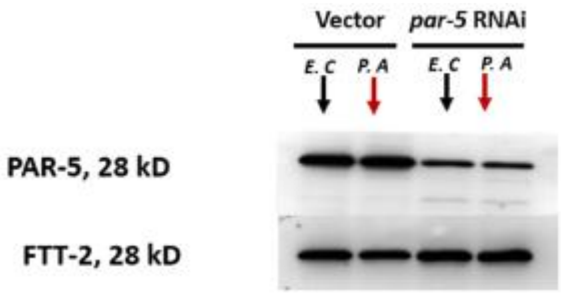

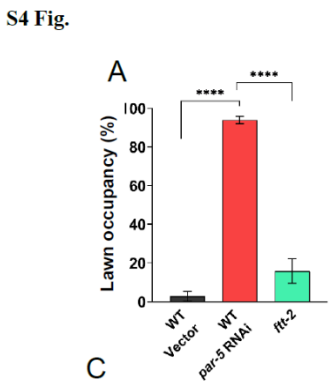

As explained in my e-mail, it is not common practice in the field to perform western blots to confirm RNAi, so we have gone beyond the standards in the field by confirming this not once but twice. For example, I just found a *C. elegans* article published in Plos Biology that uses an RNAi that can have multiple off-target effects: https://journals.plos.org/plosbiology/article?id=10.1371/journal.pbio.3000499 (vit-1 has 94% similarity with vit-2, 73% % similarity with vit-3, vit-4, and vit-5). The authors have not done any western blots or addressed RNAi efficiency in any way as standard in the field.

The western blot we performed and its quantification done in duplicate (Fig. S4B and S4C) is just confirmatory and as explained, not common practice in the field. We could just resubmit the manuscript removing those results, but we do not believe it would be necessary.

I appreciate the importance of triplicates for statistical purposes, but duplicates are perfectly acceptable in some cases. Fig S4C corresponds to a confirmatory experiment of a technique we decided to do (the 3 reviewers did not request it nor had any issue with it). The manuscript reads: "*Because par-5 and the homolog gene ftt-2 share ~78.2% sequence identity at the nucleotide level and ~85.9% sequence identity at the amino acid level [34], we studied the specificity of par-5 RNAi. First, we investigated the pathogen avoidance of ftt-2 mutants and found that unlike par-5 RNAi animals, ftt-2 animals were capable of avoiding P. aeruginosa (S4A Fig). We also investigated the expression of the two proteins using anti-FTT-2 and anti-PAR-5 antibodies and found that PAR-5 but not FTT-2 diminished upon par-5 RNAi (S4B and S4C Fig).*" As explained above, this situation can happen with almost every RNAi but, as the example showed above, even articles published in Plos Biology do not address it.
