# Supplementary material for: Microbial colonization induces histone acetylation critical for inherited gut-germline-neural signaling
Source: PLoS Biol. 2021 Mar 31;19(3):e3001169. doi: 10.1371/journal.pbio.3001169 (PMC8041202; doi:10.1371/journal.pbio.3001169)
Supplement: Attachment — Submitted filename: Review Hong and Aballay.docx [file pbio.3001169.s011.docx]

The work of Hong and Aballay aims at contributing to the elucidation of the mechanisms involved in the determination of behavior by signaling from the gut. The particular question the group is addressing is how the intestinal distention caused by bacteria *P. aeruginosa* in *C. elegans* mediates avoidance behaviors (by measuring the permanence of animals in the pathogen’s lawn). The authors find that the acetylation of lysine 8 in histone 4 in the gonad is caused by intestinal distention and is essential for both behavioral avoidance and inheritance. Authors highlight a role for the gonad in the intestinal-brain communication axis that triggers behavioral change. This is a fascinating topic of great significance for the field.

I suggest a number of issues need to be addressed:

1. There is no mention in the text of the role of histone modification and specifically of what the H4K8ac is doing transcriptionally. Also, authors do not discuss why this specific modification could be occurring compared to H3K4me3 or H3K4me1, and why were these three selected.
2. Authors used a t-test for their analysis (as mentioned in the methods section). They should instead use a one-way ANOVA with post-hoc analysis for those experiments that contain more than two conditions.
3. Figure 1C. It is really hard to see the gonad in these pictures or distinguish it from any other structure. A bright field or Nomarski picture should be provided and a marker to confirm the localization of the marks. It would be important to show whether neurons are also marked. Can authors explain why animals appear curved?
4. As in point 3, images in Figure S5 need improving. It is hard to distinguish the germline in the photos. Also, there is expression (red color) elsewhere. Which cells are those? It is important that authors show Nomarski images for those staining’s. It will really help to show an independent marker for the gonad or provide clear DAPI images.
5. Figure legends need more detail. For example: Quantification of western blots of extracts from *fer-1(b232)* animals exposed to *E. coli* (*E. C*) or *P. aeruginosa* (*P. A*). What is it that is being quantified? Fold change of what? How is this calculated? How many animals in each experiment? In the same line, this should be clarified in the figure axis (applies to all graphs with fold change). For those graphs quantifying intensity of histone acetylation, the same clarification will be important. Are those pixels? Intensity over a control?
6. In the second paragraph of the second section of results reads “As shown in Fig 2B, inhibition of *aex-5* and *eat-2* did elicit pathogen avoidance in *glp-1* animals”. What figure 2B shows is the opposite. I imagine this is a typo (an important one) given what is said afterward.
7. Figure 2C will be very hard to understand for a general audience. I suggest (as mentioned before for the other images) to include a Nomarski image. In these pictures, the *glp-1* mutants do not appear equally colonized by PA14-GFP as wild type animals. Additionally, there are other methods that more accurately measure the number of bacteria colonizing the animal intestine (CFU count for instance).
8. The experimental paradigm used in this work does not correspond to transgenerationally inherited phenomena. For an effect to be transgenerational animals should skip at least one generation of encounter to the pathogen and the following generation examined (see Rechavi’s papers for examples of transgenerational paradigms. For transgenerational effects involving pathogens see Palominos et al., 2017 or Moore et al., 2019). The effect observed here could be called intergenerational instead.
9. In the quantifications of immunofluorescence (Supplementary Figures) the n values seem to be random. For example:

S6 Fig n=4

S8 Fig. n=5

S11 Fig n=20

Are these n=4, n=5, n=20 per condition? 4, 5 or 20 on each bacterium or RNAi experiment? Or is it the total of animals screened. In any case, why are the numbers so different?

10. Other points (or simple suggestions):

- I suggest to mention throughout the text the strain of *P. aeruginosa* used is PA14.
- In the first paragraph of the Results section authors state “*P. aeruginosa* colonization specifically induces histone H4K8 acetylation in the infected animals”. In the following paragraph it is said that distention alone causes H4K8 acetylation. This apparent contradiction could be avoided by rephrasing the first sentence with “colonization” or other similar word because at that point they do not know whether it is specific to the pathogen.
- It would be nice if all graphs had a homogenous font size.
